# COVID-19 Vaccination Enhances the Immunogenicity of Seasonal Influenza Vaccination in the Elderly

**DOI:** 10.3390/vaccines13050531

**Published:** 2025-05-16

**Authors:** Engin Berber, Fani Pantouli, Hannah B. Hanley, Ted M. Ross

**Affiliations:** 1 Department of Infection Biology, Lerner Research Institute, Cleveland Clinic, Cleveland, OH 44195, USA; berbere3@ccf.org; 2 Florida Research and Innovation Center, Cleveland Clinic, Port Saint Lucie, FL 34987, USA; pantouf@ccf.org; 3 Center for Vaccines and Immunology, University of Georgia, Athens, GA 30602, USA; hhanley2@uga.edu

**Keywords:** COVID-19, elderly, influenza vaccine, mRNA vaccine

## Abstract

Background/Objectives: The co-circulation of both influenza viruses and SARS-CoV-2 poses a significant health risk, especially for the elderly. While vaccination against both diseases remains an effective strategy to reduce the burden of symptomatic infections, the effect of administering COVID-19 mRNA and seasonal influenza vaccines (COV-Flu) on elicited antibody responses has not been explored. Methods: Participants between 18 and 90 years old were vaccinated with COVID-19 mRNA vaccines (*n* = 67), seasonal influenza vaccines (*n* = 130), or both (*n* = 201) within a three-month period between 2021 and 2024. Serum hemagglutination-inhibition (HAI) titers against influenza A (H1N1, H3N2) and B (Yamagata, Victoria) strains were measured from the COV-Flu participants or the participants vaccinated with influenza vaccines only (mono-Flu). SARS-CoV-2 neutralization assays were performed on sera collected from the COV-Flu participants and the participants receiving the mRNA vaccine only (mono-COVID-19). Results: The administration of influenza virus vaccines and COVID-19 mRNA vaccines within a three-month period significantly enhanced the post-vaccination HAI titers against both influenza A and B vaccine components, particularly in the elderly (65–90) participants. There were no significant differences in SARS-CoV-2 neutralization titers in COV-Flu participants compared to mono-COVID-19 participants. Conclusions: Vaccination with both the COVID-19 mRNA and influenza vaccines enhances influenza-specific HAI titers without compromising the neutralization titers elicited by COVID-19 mRNA vaccination against SARS-CoV-2, especially in the elderly. These findings indicate the potential benefits of this approach, particularly for older adults, by boosting influenza virus vaccine-induced serum HAI activity while maintaining COVID-19 protective immunity.

## 1. Introduction

Both the SARS-CoV-2 and seasonal influenza viruses pose significant public health challenges, particularly for older adults who are at an increased risk for severe outcomes from infection [1]. COVID-19, caused by the SARS-CoV-2 virus, can lead to severe respiratory illness, long-term complications, and increased mortality, especially among individuals with comorbidities [2]. Similarly, influenza viruses are contagious respiratory pathogens that can also result in severe illness and death, particularly in young children and infants, as well as older populations [3]. Both viruses circulate all year long, but peak infections usually occur during the fall and winter months each year [4]. Co-infection with the influenza virus and SARS-CoV-2 can lead to higher rates of hospitalization and mortality compared to an infection with either virus alone [5,6].

Vaccination against both of these pathogens reduces the burden of disease following infection [7,8]. The World Health Organization (WHO) initially recommended a 14-day interval between the administration of COVID-19 vaccines and other vaccines due to the lack of evidence regarding the safety and effectiveness of co-administration [9]. However, subsequent data demonstrated that the co-administration of COVID-19 vaccines (ChAdOx1, BNT162b2, mRNA-1273, and NVX-CoV2373) with seasonal inactivated influenza vaccines had no observed adverse events compared to the administration of each vaccine separately, even though some vaccine-related reactions were reported [10,11,12,13,14]. However, few studies have examined whether receiving both vaccines affects the immune responses elicited by each vaccine. Both SARS-CoV-2 and influenza viruses elicit strong antibody-mediated immune responses, suggesting a potential cross-stimulation effect when vaccines are administered together [15]. Although co-vaccination on the same day has been explored, this study investigated the impact of administering both a COVID-19 mRNA vaccine and an influenza virus vaccine within a three-month period.

In this study, vaccine-induced antibody responses were assessed in people who were either vaccinated with only one of the vaccines or received both vaccines within a three-month period. The goal was to determine whether the administration of these vaccines within a few months enhanced or interfered with the elicited immune responses by either vaccine in young and elderly individuals. Following separated vaccination with COVID-19 mRNA and influenza vaccines within a three-month time span, the collected serum samples had higher hemagglutination-inhibition (HAI) activity against influenza vaccine components compared to the participants vaccinated only with the influenza vaccine. This phenomenon was more pronounced in the elderly compared to the younger participants. In contrast, there was no significant difference in the serum neutralization titers against SARS-CoV-2 between those who received both vaccines within a three-month period and those vaccinated with the COVID-19 mRNA vaccine only.

## 2. Materials and Methods

### 2.1. Study Design

The participants who received COVID-19 mRNA vaccines (ModernaTX, Inc., Princeton, NJ, USA or Pfizer Inc., Bio-N-Tech, New York, NY, USA) were recruited in Athens, GA, USA, from either the SARS Sero-Prevalence and Respiratory Tract Assessment (SPARTA) study [16] or the longitudinal influenza vaccine study during the 2021–2022 (UGA6), 2022–2023 (UGA7), and 2023–2024 (UGA8) seasons [17]. The participants were classified as COVID-19 monovaccinated (mono-COVID-19, *n* = 67), following vaccination with a COVID-19 mRNA vaccine only; influenza monovaccinated (Mono-Flu, *n* = 130), following the administration of FluBlok/Fluzone (Sanofi Pasteur, Swiftwater, PA, USA), Flucelvax (Seqirus Inc., Summit, NJ, USA), or Flumist (Med-Immune, Vaccines Inc., Bethesda, MD, USA) only; or covaccinated (COV-Flu, *n* = 201), following vaccination with both a COVID-19 mRNA vaccine and a seasonal influenza vaccine within a three-month period of each vaccination (Table 1). Serum samples were collected from the participants between 18 and 90 years of age from August to December each influenza vaccine season (Table 1). All the participants selected for the mono-Flu study cohorts were matched with participants who received both COVID-19 mRNA and influenza vaccines based on their age, sex, and the type of flu vaccine they received. For the mono-COVID-19 cohorts, participants were selected to match the age and sex characteristics among the mRNA COVID-19 vaccine recipients (Supplementary Table S1). All the mRNA vaccine recipients completed their primary series of vaccination and were boosted with an mRNA COVID-19 vaccine before joining the study. All the vaccine participants were further categorized into two groups: young adults aged 18–64 years and elderly individuals aged 65–90 years.

### 2.2. Patient Consent Statement

Informed and written consent was obtained from all the participants prior to their inclusion in the Seroprevalence and Respiratory Tract Assessment (SPARTA) study [16], as well as in studies evaluating immune responses and comparing influenza vaccines conducted at the University of Georgia Athens (UGA) in Athens, GA, USA. The study procedures involved collecting demographic information and collecting blood cell, serum, and saliva samples as reviewed and approved by the Institutional Review Board of WIRB-Copernicus Group Institutional Review Board [WCG IRB #20202906 for SPARTA and WCG IRB# 20224877 for UGA] and by the University of Georgia Institutional Review Board. All the participants and samples were de-identified and assigned an alphanumeric code to ensure participant confidentiality. The study adhered to ethical guidelines and regulations in accordance with the Helsinki Declaration and other applicable principles for research involving human participants.

### 2.3. Vaccinees and Formulations

The participants who received a COVID-19 vaccine received either the Moderna (mRNA-1273) or the Pfizer (BNT162b2) mRNA vaccine. Those enrolled during the 2021 vaccination season received a monovalent mRNA vaccine formulated with the ancestral Wuhan SARS-CoV-2 strain. The participants enrolled during the 2022–2023 season received a bivalent COVID-19 vaccine containing both the ancestral strain and Omicron BA.4/BA.5 variants, as well as the monovalent (XBB.1.5) vaccine during the 2023–2024 season.

The participants who were vaccinated with seasonal influenza virus vaccines received a quadrivalent influenza vaccine (QIV) containing two influenza A virus (IAV) strains (A/H1N1 and A/H3N2) and two influenza B virus (IBV) strains (Yamagata and Victoria lineages). The participants who were 65–90 years old received the Fluzone high dose (HD), and the participants who were 18–64 years old received Fluzone standard dose (SD) or FluBlok (Supplementary Table S1).

### 2.4. Hemagglutination Inhibition (HAI) Assay

All the serum samples were assessed using the validated hemagglutinin-inhibition (HAI) assay [18] and were tested, as previously described [17,19], against A/H1N1, A/H3N2, B/Yamagata, and B/Victoria strains per season. The serum samples were treated with three volumes of receptor-destroying enzyme (RDE) (Denka, Seiken, Co., Tokyo, Japan) overnight at 37 °C, followed by heat inactivation at 56 °C for 30 min. The sera samples were then diluted 1:10 by adding six volumes of 1× phosphate-buffered saline (PBS) (Corning, Manasses, VA, USA) to the final solution. In a 96-well, U-bottom microtiter plate (Thermo Fisher, Waltham, MA, USA), two-fold serial dilutions of the heat-inactivated sera were prepared. An equal volume of a working solution of the virus, containing 8 hemagglutination units (8 HAU), was prepared by diluting the viral stock in PBS. Serially diluted sera samples were mixed with the virus solution in each well, and the plate was incubated at room temperature for 20 min to facilitate virus–antibody interactions. Following incubation, 0.8% turkey red blood cells (RBCs) (Lampire Biologicals, Pipersville, PA, USA), diluted in PBS, were added to the wells. The plates were covered and incubated at room temperature for 30 min. HAI assays against H3N2 viruses were performed using guinea pig red blood cells (Lampire Biologicals, Pipersville, PA, USA) at a final concentration of 0.75%, supplemented with 20 nM oseltamivir, a neuraminidase inhibitor. Hemagglutination patterns were assessed visually by tilting the plates. The hemagglutination inhibition titer was recorded as the reciprocal of the highest serum dilution that completely inhibited hemagglutination and compared to the sera reference controls included on each plate. The HAI titers were defined according to the Committee for Proprietary Medicinal Products (CPMP) criteria. Those HAI titers that were lower than 40 (HAI titer < 40) were accepted as “seronegative”, while HAI titers that were equal to or over 40 (HAI titer ≥ 40) were accepted as “seropositive”. Seroconversion was identified if a pre-vaccination serum HAI titer measured negative, and then the post-vaccination titer measured on day 28 was ≥40 and demonstrated a 4-fold increase compared to the pre-vaccination HAI titer calculated [20].

### 2.5. SARS-CoV-2 Serum Neutralization Assay

The serum samples were tested in a SARS-CoV-2 neutralization assay, as described before [21]. Replication-defective lentiviral particles expressing green luciferase (BEI Resources, www.beiresources.org) were pseudotyped with the SARS-CoV-2 spike glycoprotein and were used to measure neutralizing antibody activity. The pseudotyped virus was propagated using HEK293T cells. Co-transfection was performed to generate pseudotyped viruses. For each T150 flask, 6.64 µg of the Wuhan-Hu-1 SARS-CoV-2 spike glycoprotein-expressing plasmid (NR-52514) was mixed with 4.3 µg each of the helper plasmids expressing Gag; pol (HDM-Hgpm2; NR-52517), Rev1b (pRC-CMV-Rev1b; NR-52519), and Tat1b (HDM-tat1b; NR-52518), along with 19.5 µg of the Luciferase-IRES-ZsGreen backbone (NR-52516) in 1.95 mL Opti-MEM I serum-reduced medium (Life Technologies, Grand Island, NY, USA) containing 78 µL P3000 reagent (Invitrogen, Carlsbad, CA, USA). The P3000 and Lipofectamine 3000 (Invitrogen, Carlsbad, CA, USA) was used for the transfection of the plasmids. The flask was incubated in a 37 °C cell culture incubator for 72 h. Virus-containing cell supernatants were collected and centrifuged at 2100× *g* rpm for 10 min. The supernatant was filtered using a 0.45 µm filter (Thermo Fisher, Rochester, NY, USA). The filtrate was then concentrated using Amicon Ultra-15, 100 K (Merck Millipore, Tullagreen, Carrigtwohill Co Cork, IRL) following the manufacturer’s protocol.

HEK293-ACE2 cells (BEI resources) were seeded with a 4.6 × 10^5^ cell/well density. The concentrated virus was serially diluted (3-fold) in 2% FBS containing DMEM media supplemented with 1% Pen-Strep antibiotic and incubated at 37 °C. After 48 h of incubation, the cells were treated with Bright-GLO substrate (Promega, Madison, WI, USA) and then incubated for 5 min at RT. The lysed cells were transferred to white, opaque, 96-well plates before reading the luminescence activity on a GloMax Discover microplate reader (Promega, Madison, WI, USA). Relative Luciferase Units (RLUs) corresponding to the virus dilution yielded a >1000-fold RLU signal (minimum 4 × 10^6^ RLU/mL) compared to when a cell-only background was selected. Relative Luciferase Unit (RLU) values were used to calculate the pseudovirus neutralization titers at 50% (PsVN) and their respective serum dilutions through a nonlinear regression analysis of the inhibitor concentration versus the normalized response to obtain the inhibitor concentration 50 (IC50) values by using GraphPad Prism Version 10 Software (GraphPad Software Inc., San Diego, CA, USA). The RLUs were normalized based on controls for both virus-only and cell-only conditions. Specifically, the mean RLU of the cell-only control was defined as representing 100% neutralization, whereas the mean RLU from the virus-only control was considered as 0% neutralization. The IC50 values correspond to PsVN for the tested serum samples, with the results summarized as geometric mean titers (GMTs) with their standard deviation (SD). The samples that were unable to neutralize SARS-CoV-2 at dilutions of less than 1:20 were assigned a threshold value of 20 for subsequent analyses [22].

### 2.6. Statistical Analysis

The statistical significance of the differences in HAI titers and neutralization titers between two groups was calculated using a two-tailed, unpaired, non-parametric Mann–Whitney test. Fisher’s exact test was used to compare the categorical variability between seroconverters and non-seroconverters. GraphPad Prism 10 software (GraphPad, San Diego, CA, USA) was used for the statistical analysis and the data visualization. For this study, a *p* value of ≤ 0.05 was defined as being statistically significant (* *p* <0.05, ** *p* < 0.01, *** *p* < 0.001, **** *p* < 0.0001).

## 3. Results

### 3.1. Demographics of Participants

The participants vaccinated with both a COVID-19 mRNA vaccine and a seasonal influenza vaccine within a three-month period were classified as COV-Flu (*n* = 201). The participants who received only the COVID-19 mRNA vaccines were classified as mono-COVID-19 (*n* = 67), and the participants who received only the seasonal influenza vaccine were classified as mono-Flu (*n* = 130) (Table 1). All the participants were matched based on their demographic characteristics. When all the cohorts were combined, the average age of the young adults was ~44 years of age, while the older adults (elderly) averaged ~72 years of age. Most of the participants were white (>80%) and female (Table 1). For the participants receiving COVID-19 mRNA, the majority received the Pfizer COVID-19 mRNA vaccine, while the remaining participants received the Moderna mRNA vaccine (Supplementary Tables S1 and S2). For influenza, the vaccine choices varied by the age group and the season (Supplementary Table S1).

### 3.2. Comparison of HAI Titers Between COV-Flu and Mono-Flu Participants

**Figure 1:**
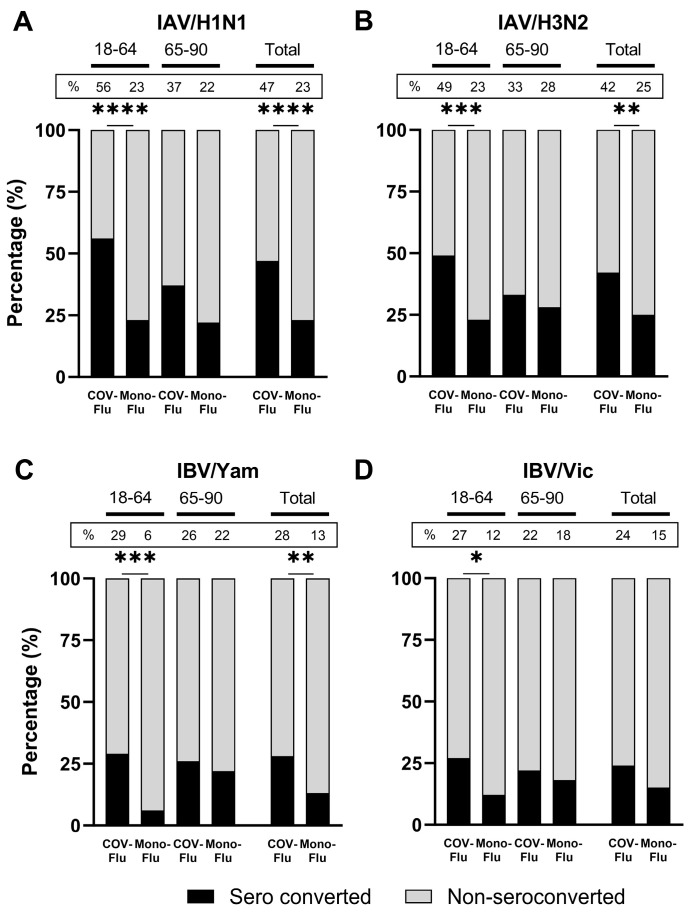
Comparison of strain-specific HAI seroconversion against influenza strains between COV-Flu and mono-Flu individuals. The panels show the percentage (%) of seroconverters and non-seroconverters against A/H1N1 (**A**), A/H3N2 (**B**), B/Yamagata (**C**), and B/Victoria (**D**) during the UGA6 (2021–2022), UGA7 (2022–2023), and UGA8 (2023–2024) seasons combined among the participants who received Fluzone and FluBlok vaccines. The percentage of seroconverters is displayed at the top of the bars. Seroconverters were defined as individuals with a four-fold rise in the HAI titer, to ≥40, by day 28. The number of participants in COV-Flu (18–64 y.o.), *n* = 97; in mono-Flu (18–64 y.o.), *n* = 65; in COV-Flu (65–90 y.o.), *n* = 87; and in mono-Flu (65–90 y.o.), *n* = 50. The total number of COV-Flu participants was *n* = 184, and the total number of mono-Flu participants was *n* = 115. Fisher′s exact test was performed to compare significant associations between COV-Flu and mono-Flu participants (* *p* < 0.05, ** *p* < 0.01, *** *p* < 0.001, **** *p* < 0.0001).

Overall, there were significantly higher rates of seroconversion against each influenza vaccine component in all the COV-Flu adult participants compared to participants administered only the influenza vaccine (mono-Flu) (Figure 1A–D). However, in the elderly participants, there was no statistical difference in the seroconversion rates between the COV-Flu and the mono-flu participants against the IAV or IBV components (Figure 1A–D).

**Figure 2:**
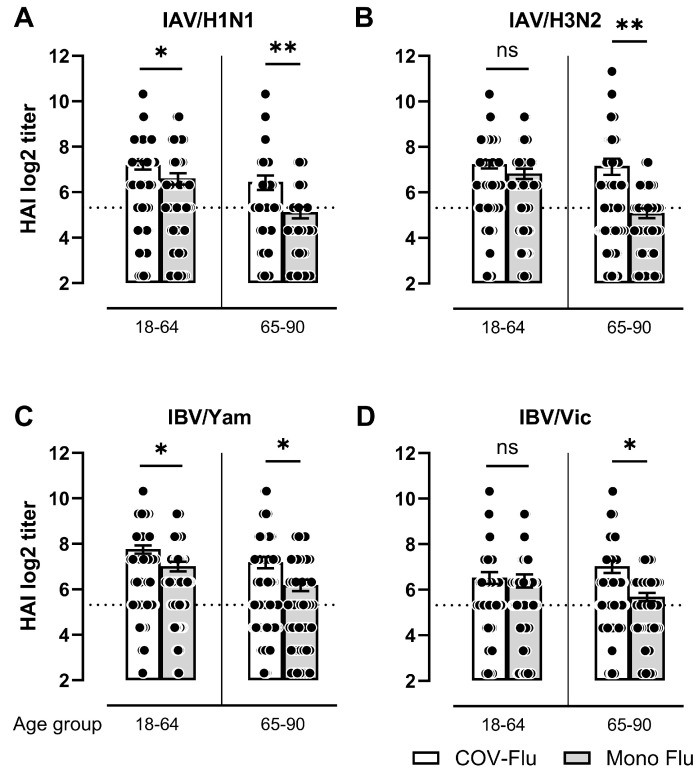
Comparison of HAI (log2) titers against influenza vaccine strains during 2021–2024 vaccination seasons (UGA6-7-8). Individuals vaccinated with COVID-19 mRNA and influenza vaccines (Fluzone and FluBlok) within a three-month period (COV-Flu) were compared to mono-Flu (Fluzone and FluBlok)-vaccinated participants among young-adult (18–64) and elderly participants (65–90). HAI responses shown against A/H1N1 (**A**), A/H3N2 (**B**), B/Yamagata (**C**), and B/Victoria (**D**). Numbers of participants in COV-Flu (18–64 y.o.), *n* = 97; in mono-Flu (18–64 y.o.), *n* = 65; in COV-Flu (65–90 y.o.), *n* = 87; and in mono-Flu (65–90 y.o.), *n* = 50. The dotted line represents the seropositivity limit of log2 40 HAI. A non-parametric Mann–Whitney test was performed comparing two groups (* *p* < 0.05, ** *p* < 0.01, ns: not significant).

The participants who were vaccinated with a COVID-19 mRNA vaccine and an influenza vaccine (COV-Flu) had, in general, higher average HAI titers against each of the four vaccine components compared to the participants who only received the influenza vaccine (mono-Flu) (Figure 2A–D). Against IAV strains, sera collected from the elderly participants had significantly higher HAI titers (*p* < 0.01) compared to sera from the elderly participants vaccinated with only influenza virus vaccine, whereas it was significant only against IAV/H1N1 in the young adults (*p* < 0.05). Sera collected from both the young-adult and elderly COV-Flu participants had significantly higher titers against IBV/Yam (*p* < 0.05) (Figure 2C). In contrast, only sera collected from the COV-Flu elderly participants had significantly higher HAI titers (*p* < 0.05) against IBV/Vic (Figure 2D).

**Figure 3:**
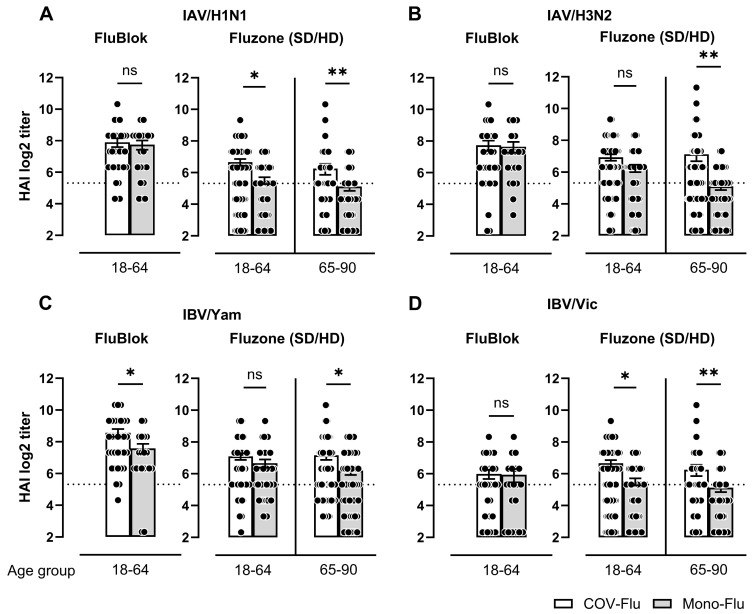
Comparison of HAI titers by influenza vaccine types in the young-adult (18–64) and elderly (65–90) participants. HAI titers represented for FluBlok and Fluzone SD/HD-type vaccines administered during UGA6 (2021–2022), UGA7 (2022–2023), and UGA8 (2023–2024) vaccination seasons. The HAI titers are shown against the influenza A viruses (IAV); H1N1 (**A**) and H3N2 (**B**), and influenza B viruses (IBV); Yamagata (**C**) and Victoria (**D**), virus components in COV-Flu- and mono-Flu-vaccinated participants for FluBlok and Fluzone standard dose (SD) and high dose (HD) vaccine recipients. Numbers of participants in COV-Flu with FluBlok (18–64 y.o.), *n* = 32; in mono-Flu (18–64 y.o.), *n* = 20; for Fluzone SD in COV-Flu (18–64 y.o.), *n* = 65; in mono-Flu (18–64 y.o.), *n* = 45; in COV-Flu (65–90 y.o.), *n* = 80; and in mono-Flu (65–90 y.o.), *n* = 50. The dotted line represents the seropositivity limit of log2 40 HAI. A non-parametric Mann–Whitney test was performed comparing two groups (* *p* < 0.05, ** *p* < 0.01, ns: not significant).

Similar results were observed when the participants were classified as being between the ages of 18 and 49 years old or 50 and 90 years old (Supplementary Figure S1). Vaccination with any of the four vaccines (Fluzone, FluBlok, Flucelvax, or Flumist) yielded similar results, except against IAV/H3N2 in young adults, where a significant difference (*p* < 0.05) was observed between the COV-Flu and the mono-Flu participants. This difference was noted in the Flucelvax participants when analyzed by the vaccine type, with higher HAI titers in the COV-Flu participants compared to the mono-Flu participants (Supplementary Figures S2 and S3). Most of the participants were vaccinated with either the FluBlok or the Fluzone (SD/HD) influenza virus vaccines. In the 18–64 age group, there were no significant differences in serum HAI titers between the covaccinated participants and the participants receiving only the FluBlok against the H1N1 and H3N2 components (Figure 3A,B). Sera from the covaccinated participants vaccinated with Fluzone had significantly higher HAI titers against all four components in the elderly participants compared to the influenza-only monovaccinated participants (Figure 3A–D). Sera from the covaccinated young-adult participants had significantly higher HAI titers following vaccination with Fluzone against the H1N1 and B/Vic components (Figure 3 A,D).

### 3.3. SARS-Cov-2 Virus Neutralization Titer

**Figure 4:**
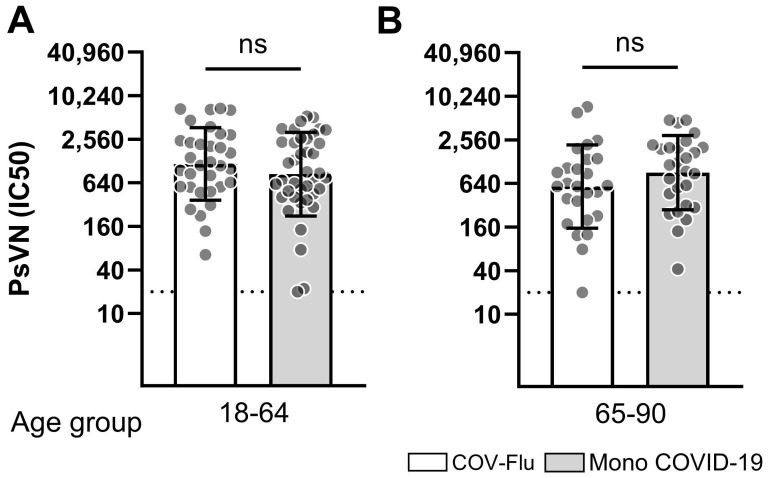
Comparison of pseudovirus neutralization titers against SARS-CoV-2 Wuhan Hu-1. Pseudovirus neutralization (PsVN) titers were shown with an inhibitor concentration of 50 (IC50) in individuals vaccinated against COVID-19 and influenza (COV-Flu) versus those vaccinated only with the COVID-19 mRNA vaccine (mono-COVID-19) who were young-adult (18–64) (**A**) or elderly participants (65–90) (**B**). The number of participants in COV-Flu (18–64), *n* = 33; in mono-COVID-19 (18–64), *n* = 40; in COV-Flu (65–90), *n* = 26; in mono-COVID-19 (65–90), *n* = 27. Geometric mean titers (GMTs) with standard deviations (SDs) are presented for each group. The dotted line indicates the assay threshold, which is the starting serum dilution, 1:20. PsVN titers lower than 20 IC50 are normalized to 20. A non-parametric Mann–Whitney test was performed comparing two groups (ns, *p* > 0.05).

Sera collected from all the participants who received an mRNA COVID-19 vaccination had neutralization activity against the SARS-CoV-2 ancestral Wuhan-Hu-1 strain (Figure 4 and Supplementary Table S3). Sera collected from the COV-Flu participants had statistically similar neutralization titers compared to the participants who received only the COVID-19 vaccination (Figure 4A,B). Similar titers were obtained from all the age groups.

## 4. Discussion

In this study, participants who enrolled in an influenza vaccine study during the 2021–2022 (UGA6), 2022–2023 (UGA7), and 2023–2024 (UGA8) influenza seasons were vaccinated with one of four different influenza vaccines. Many of the participants also received the COVID-19 mRNA vaccine during this time period. Therefore, the goal of this study was to assess the influence of vaccination on the elicited immune responses in these participants who received influenza virus vaccines with or without vaccination with COVID-19 mRNA vaccines. The participants vaccinated with both influenza and COVID-19 mRNA vaccines had, in general, higher HAI activity against the influenza vaccine components in three consecutive seasons during the COVID-19 pandemic compared to the participants who received only the influenza virus vaccine. In contrast, the participants vaccinated with both vaccines did not have enhanced neutralizing titers against SARS-CoV-2. The administration of the COVID-19 mRNA vaccine and the influenza vaccine on the same day has not been shown to significantly increase adverse events [13]. The participants who received both vaccines concurrently maintained a similar reactogenicity profile as the participants vaccinated with either vaccine alone [10,11,12,13].

These enhanced influenza immune responses were more prevalent in the elderly participants (65–90 y.o.) than the young-adult participants (18–64 y.o.). The Centers for Disease Control and Prevention (CDC) and the Advisory Committee on Immunization Practices (ACIP) classify individuals aged 65 years and older as being at a higher risk for serious complications of influenza, and they recommend the use of higher-dose or adjuvanted flu vaccines [23,24]. In general, elderly participants have a more rapid decline in anti-influenza antibodies following vaccination each season, and many return to a baseline 12 months after vaccination [25]. Annual vaccination is necessary for older participants whose antibody titers tend to decline to non-protective levels from one year to the next. This allows for higher rates of seroconversion following influenza virus vaccination each season, since most younger adults can retain antibody titers from season to season [17,25]. Young-adult participants who receive influenza virus vaccines in two or more consecutive seasons are more likely to be seropositive at D0 against all four vaccine components compared to elderly participants [17]. Elderly participants have the capacity to increase antibody titers from a lower baseline compared to young-adult participants who enter an influenza season with higher antibody titers. Elderly participants have more immunological space in which to observe a rise in antibody titers, since the initial HAI titer is at a lower baseline level than the young-adult participants. Similarly, the enhanced HAI titers against influenza viruses were more prevalent in the elderly participants who received both COVID-19 mRNA and influenza vaccines compared to the young-adult participants, which may be associated with the ability to observe larger rises in seroconversion and HAI activity compared to young adults. Only a few studies have investigated the immunogenicity of vaccination on the same day with COVID-19 mRNA and influenza virus vaccines. Participants who received both COVID-19 mRNA and influenza virus vaccines in the same arm concurrently had a significantly higher HAI antibody titer against the H3N2 influenza virus component in the vaccine compared to the HAI response in participants who received these same vaccines in different arms [26]. In contrast, other studies reported that there were no significant differences in serum HAI titers against influenza vaccine components following same-day vaccination [27] or antibody binding titers against the spike protein of the SARS-CoV-2 virus [28]. Such differences between different studies may be attributed to variations in the study populations, including a wide age range of participants and differences in the types of influenza vaccines administered.

In general, FluBlok elicited higher HAI titers against the IAV and IBV strains compared to Fluzone in vaccinated adults. FluBlok generally elicited higher HAI titers, particularly against H1N1 influenza viruses, compared to Fluzone in adults [29]. FluBlok is composed of recombinant HA proteins representing H1, H3, B/Vic, and/or B/Yam and efficiently elicits anti-HA antibody responses. Fluzone is a split, inactivated virus vaccine containing a mix of viral proteins other than HA, such as NP and M1, which might dilute the immune focus on HA. Vaccination with both COVID-19 mRNA and flu vaccines (either FluBlok or with Fluzone) within a three-month period elicited higher HAI titers than just influenza vaccination alone in younger adults. The effects were more pronounced in the elderly participants than the young-adult participants who received Fluzone. Since FluBlok is approved for people aged 60 years or younger, few elderly participants were vaccinated with FluBlok, and no elderly participants were vaccinated with the Flumist or Flucelvax vaccines.

Lipid nanoparticles (LNP), used in the formulation of COVID-19 mRNA vaccines, may act as an adjuvant by stimulating innate immunity [30]. LNPs activate toll-like receptors (TLR) 4 and TLR7/8, thereby promoting the activation of dendritic cells (DCs) and the production of cytokines, such as IL-6 and interferon-gamma [31]. The formulation of LNP influenza recombinant hemagglutinin protein has been shown to elicit stable HAI titers for at least 20 weeks in mice after a single immunization, which outperformed an MF59-adjuvant-formulated vaccine. Furthermore, and LNP-formulated influenza vaccine induced the stimulation and activation of antigen-specific CD4+ T follicular helper cells and germinal center B cells via IL-6 cytokine induction from lymph nodes [32]. LNPs alone, with no antigen formulation, elevate the activation and maturation of antigen-presenting dendritic cells in vitro when healthy PBMCs are stimulated, leading to the increased production of IL-6, IL-12, and IL-21 and IFN-γ. Notably, stronger responses are observed in younger individuals compared to the elderly [30]. The immune response elicited by LNP adjuvants appears to affect antigen-specific immunity by enhancing both B cell and antigen-specific T cell responses, which are components of the trained immune response [33]. Therefore, the mRNA LNP vaccine, when administered relatively close in time with an influenza vaccine, may provide an adjuvant effect to enhance anti-influenza virus immunity. FluAd is an MF59-adjuvant-formulated influenza virus vaccine that enhances HAI titers, particularly against H3N2 and IBV strains [34], that is approved for use in the elderly [35]. Studies have indicated that MF59 can lead to an increase in germinal center B cell differentiation and persistence for up to four months post-immunization [36]. Although MF59 is known to induce adaptive immune responses by recruiting immune cells to the injection site [35], a more efficient and durable generation of germinal center B cells and memory B cell responses has been observed with the LNP-formulated influenza vaccine in vivo compared to the MF59-formulated vaccine [32]. Therefore, the effect of COVID-19 mRNA and flu vaccination may mimic the use of adjuvants with influenza vaccines.

The enhancement of immune responses that was observed in the participants vaccinated with both COVID-19 mRNA and flu vaccines was specific to the antibodies elicited by influenza vaccines and not by the antibodies elicited by the COVID-19 mRNA vaccines. COVID-19 mRNA vaccination resulted in the neutralization titer against the ancestral Wuhan-Hu-1 strain in the majority of the participants. The neutralization titers in both young-adult and elderly participants who received both vaccines were statistically similar to those in the participants vaccinated only with the COVID-19 mRNA vaccine.

## 5. Conclusions

Vaccination with both a COVID-19 mRNA LNP vaccine and an influenza virus vaccine within a three-month period enhanced the HAI antibody responses elicited by the influenza virus vaccine, particularly in the elderly participants. The observed increase in HAI titers across multiple influenza seasons that were observed with different types of influenza virus vaccines indicate the potential benefit of vaccination with both COVID-19 mRNA and flu vaccines within a three-month period, as covaccination boosted the immune response against seasonal influenza viruses. Since co-infections with SARS-CoV-2 and influenza viruses can increase the severity of illness, administering both COVID and influenza vaccines within a three-month period may provide added protection against influenza virus infections in the elderly.

## Figures and Tables

**Table 1:** Demographic characteristics of study group participants during 2021–2024 from UGA-6, -7, and -8 influenza vaccine study cohorts.

		UGA6 (2021–2022) ^a^		UGA7 (2022–2023)		UGA8 (2023–2024)		Total UGA6-8 (2021–2024)		SPARTA (2021–2024) ^b^
Demographic Characteristics		COV-Flu ^c^ (*n* = 109)	Mono-Flu ^d^ (*n* = 56)	COV-Flu (*n* = 61)	Mono-Flu (*n* = 48)	COV-Flu (*n* = 31)	Mono-Flu (*n* = 26)	COV-Flu (*n* = 201)	Mono-Flu (*n* = 130)	Mono-COVID-19 ^e^ (*n* = 67)
Sex (%)										
	Male	37 (34%)	16 (29%)	28 (46%)	21 (44%)	9 (29%)	11 (42%)	74 (37%)	48 (37%)	23 (34%)
	Female	72(66%)	40 (71%)	33 (54%)	27 (56%)	22 (71%)	15 (58%)	127 (63%)	82 (63%)	44 (66%)
Age group (%)										
	Young adult (18–64)	66 (61%)	41 (73%)	30 (49%)	23 (48%)	18 (58%)	16 (62%)	114 (57%)	80 (62%)	40 (60%)
	Elderly (65–90)	43 (39%)	15 (27%)	31 (51%)	25 (52%)	13 (42%)	10 (38%)	87 (43%)	50 (38%)	27 (40%)
Average Age (age range)										
	Young adult (18–64)	39.3 (18–63)	39.7 (18–64)	44.0 (18–63)	45.2 (19–63)	48.3 (25–64)	44.9 (25–64)	43.9 (18–64)	43.3 (18–64)	44.3 (19–64)
	Elderly (65–90)	72.2 (65–86)	71.3 (65–83)	71.8 (65–87)	72.0 (66–84)	71.7 (65–80)	71.1 (65–76)	71.9 (65–87)	71.5 (65–84)	73.3 (65–83)
Race/Ethnicity (%)										
	White	94 (86%)	45 (80%)	59 (97%)	42 (88%)	28 (90%)	25 (96%)	181 (90%)	112 (86%)	60 (90%)
	Black	6 (6%)	2 (4%)	-	2 (4%)	2 (7%)	-	8 (4%)	4 (3%)	-
	Hispanic or Latino	7 (6%)	4 (7%)	2 (3%)	3 (6%)	1 (3%)	-	10 (5%)	7 (5%)	2 (3%)
	Asian	2 (2%)	3 (5%)	-	1 (2%)	-	1 (4%)	2 (1%)	5 (4%)	5 (7%)
	American Indian or Alaska Native	-	1 (2%)	-	-	-	-	-	1 (1%)	-
	Mixed (Black, White, Hispanic, or Latino)	-	1 (2%)	-	-	-	-	-	1 (1%)	-
Average duration (days) between mRNA COVID-19 vaccine dose and influenza vaccine (day range)		20.3 (1–88)	-	27.6 (1–81)	-	28 (2–87)	-	24.6 (1–88)	-	-
Average days post-flu vaccination for sera testing (day range)		29.2 (21–42)	29.6 (24–42)	28 (21–40)	28.6 (24–41)	28.9 (23–37)	28.7 (21–35)	28.8 (21–42)	29.1 (21–42)	-
Average days post-COVID-19 vaccination for sera testing (day range)		35.9 (7–87)	-	48.3 (3–88)	-	21.5 (4–78)	-	37.5 (3–88)	-	46.3 (7–90)

^a^ University of Georgia, Athens (UGA). ^b^ Seroprevalence and Respiratory Tract Assessment (SPARTA). ^c^ COVID-19 and flu vaccination within a three-month period (COV-Flu). ^d^ Influenza monovaccinated (mono-Flu). ^e^ COVID-19 monovaccinated (mono-COVID-19).

## Data Availability

The data are provided in the manuscript, and additional details and data are enclosed within Supplementary Materials.

## Supplementary Materials

The following supporting information can be downloaded at https://www.mdpi.com/article/10.3390/vaccines13050531/s1. Figure S1: Comparison of HAI (log2) titers between COV-Flu and mono-Flu participants across different age groups, stratified into 18–49 and 50–90 years; Figure S2: Comparison of HAI (log2) titers between COV-Flu and mono-Flu participants across different age groups vaccinated with Fluzone, FluBlok, Flucelvax, and Flumist; Figure S3: Comparison of HAI (log2) titers between COV-Flu and mono-Flu participants vaccinated with Flucelvax and/or Flumist influenza vaccine in adults (18–64); Table S1: The numbers and percentages of participants by vaccine type across three consecutive seasons included in the study groups; Table S2: Hemagglutination inhibition (HAI) titers against various influenza virus strains in participants; Table S3: Pseudovirus neutralization titers against the Wuhan Hu-1 variant of SARS-CoV-2.
