# Supplementary material for: COVID-19 Vaccination Enhances the Immunogenicity of Seasonal Influenza Vaccination in the Elderly
Source: Vaccines (Basel). 2025 May 16;13(5):531. doi: 10.3390/vaccines13050531 (PMC12116172; doi:10.3390/vaccines13050531)
Supplement: Supplementary file 1 [file vaccines-13-00531-s001.zip › vaccines-3627184-supplementary.pdf]

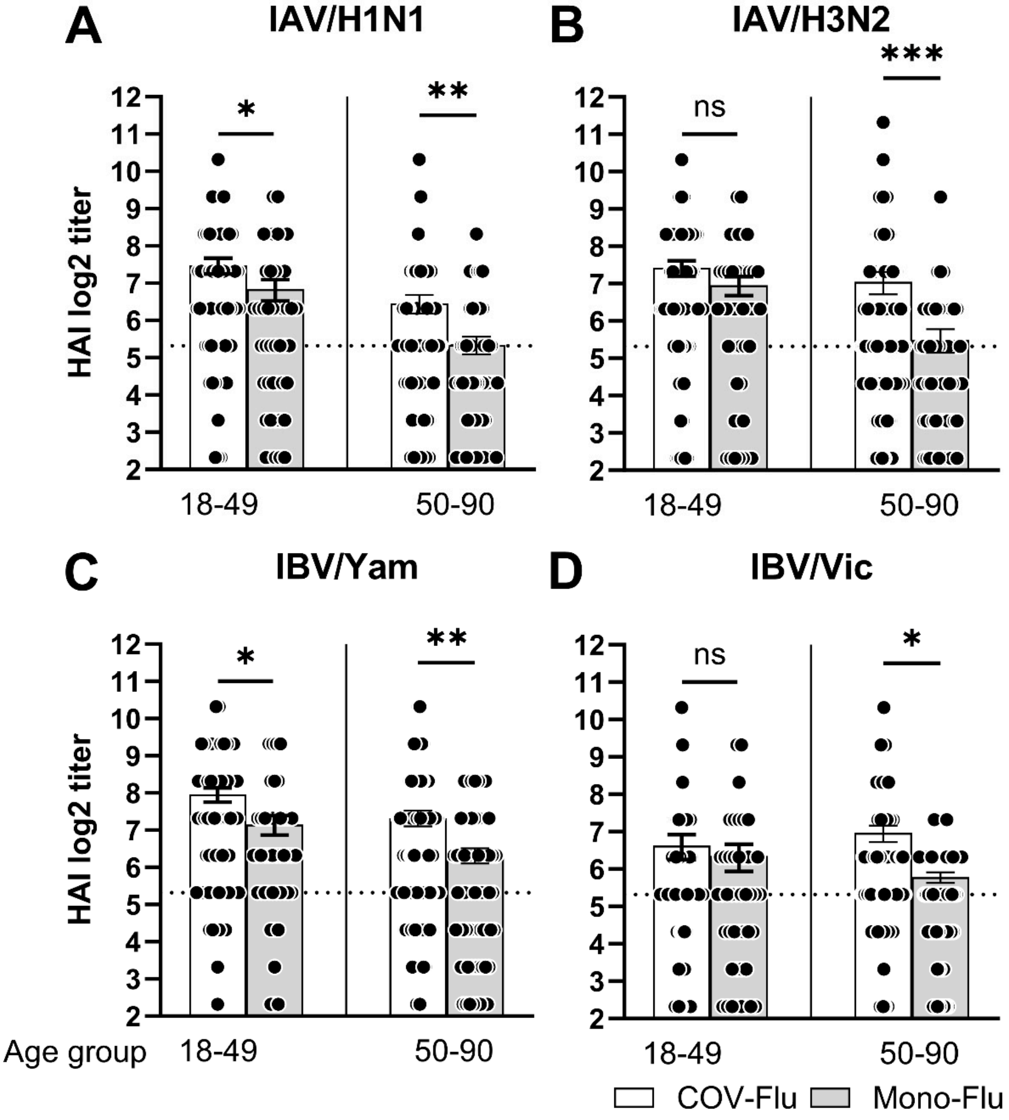

Supplementary Figure S1. Comparison of HAI (log2) titers between COV-Flu and mono-Flu participants across different age groups, stratified into 18-49 and 50-90 years. The titers shown against influenza A (IAV/H1N1) (*A*) and IAV/H3N2) (*B*) and influenza B (IBV/Yamagata) (*C*) and IBV/Victoria) (*D*) virus components in COV-Flu and mono-Flu vaccinated participants. Numbers of participants in COV-Flu (18-49 y.o.), n=69; in mono-Flu (18-49 y.o.), n=46; in COV-Flu (50-90 y.o.), n=115; and in mono-Flu (50–90 y.o.), n=69. The dotted line represents the seropositivity limit of log2 40 HAI. A non-parametric Mann-Whitney test have been performed comparing two groups (*p<0.05, **p<0.01, ***p<0.001, ns: not significant).

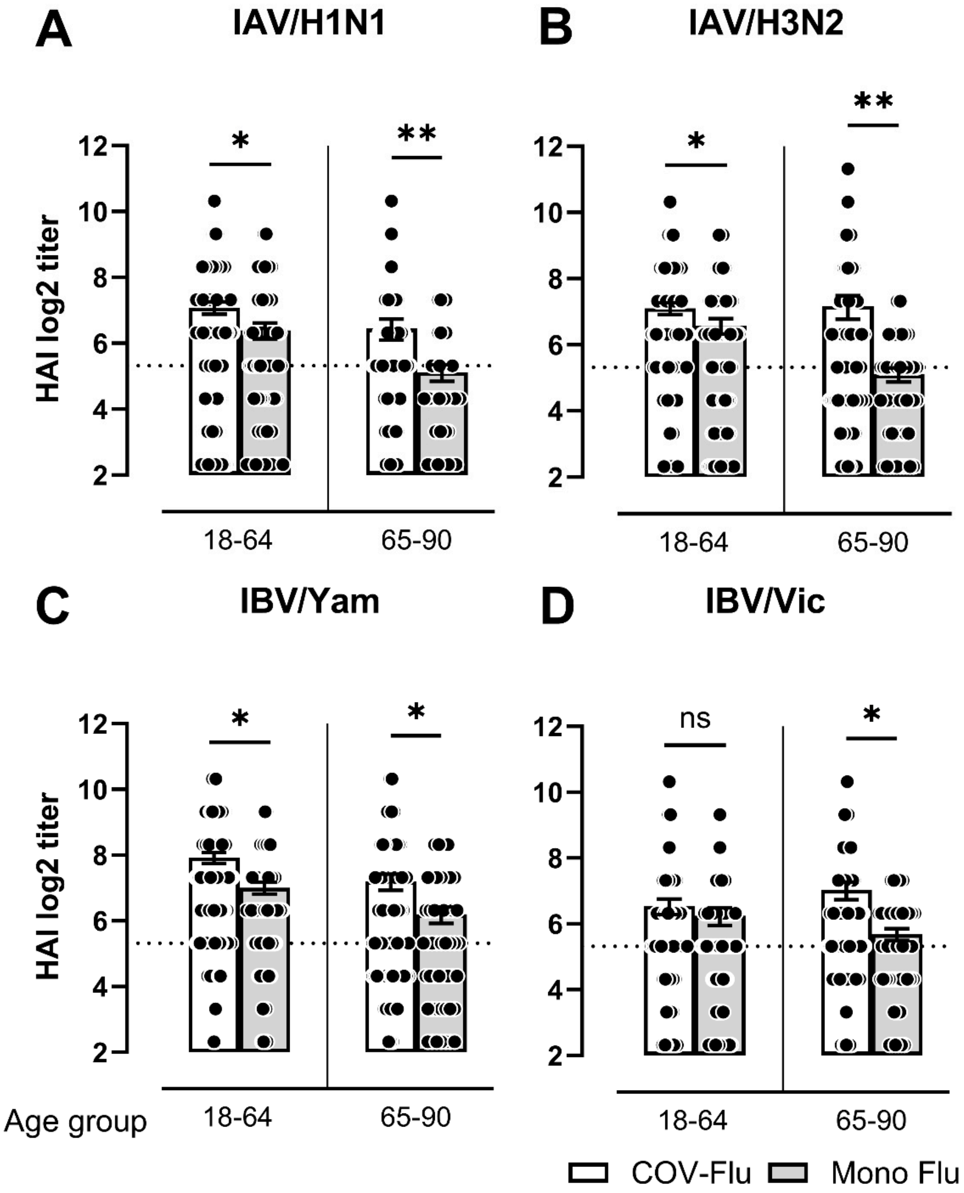

Supplementary Figure S2. Comparison of HAI (log2) titers between COV-Flu and mono-Flu participants across different age groups vaccinated with Fluzone, FluBlok, Flucelvax, and Flumist. The titers shown against influenza A (IAV/H1N1) (*A*) and IAV/H3N2) (*B*) and influenza B (IBV/Yamagata) (*C*) and IBV/Victoria) (*D*) virus components in COV-Flu and mono-Flu vaccinated participants. Numbers of participants in COV-Flu (18-64 y.o.), n=114; in mono-Flu (18-64 y.o.), n=80; in COV-Flu (65-90 y.o.), n=87; and in mono-Flu (65-90 y.o.), n=50. The dotted line represents the seropositivity limit of log2 40 HAI. A non-parametric Mann-Whitney test have been performed comparing two groups (*p<0.05, **p <0.01, ns: not significant).

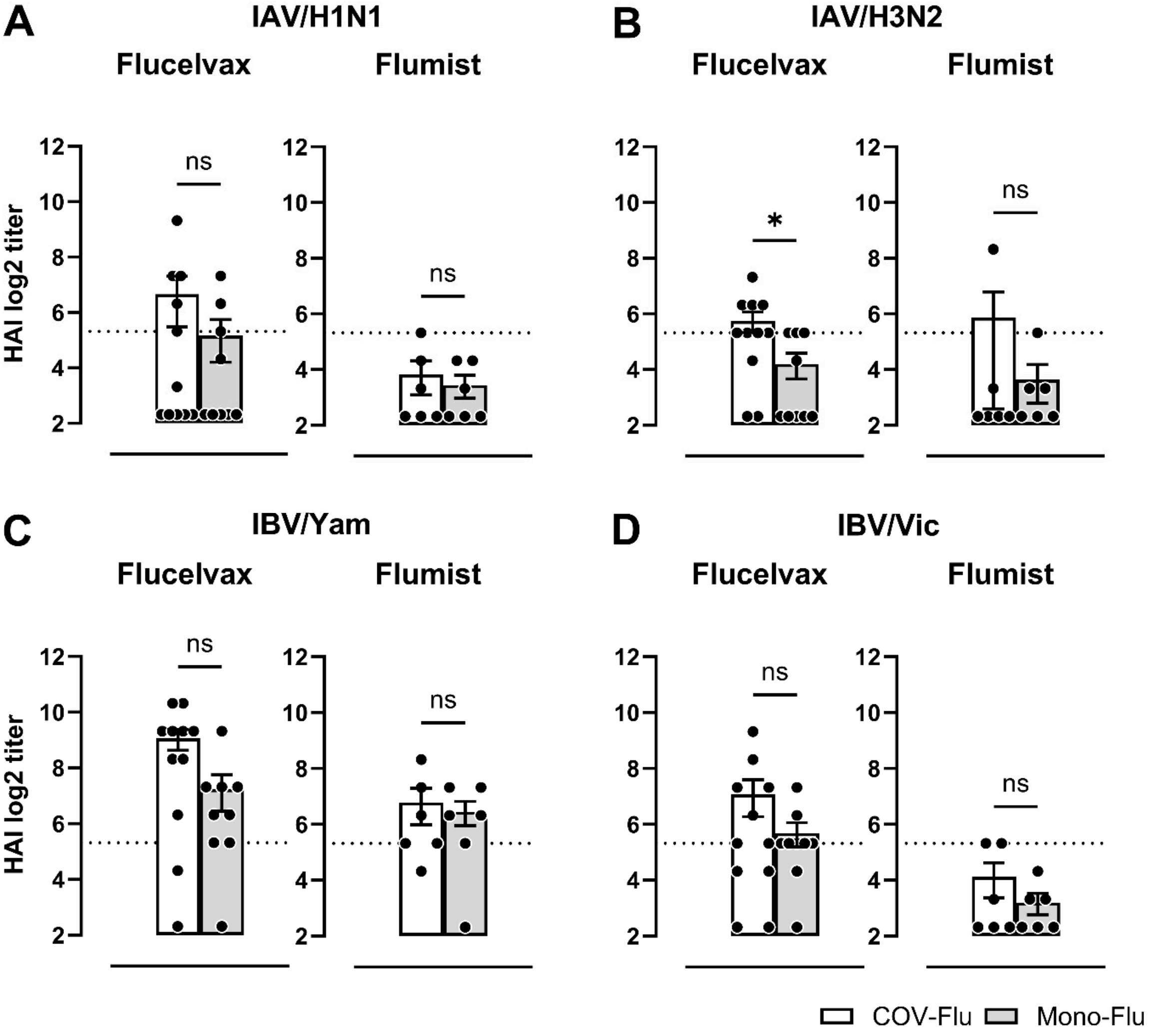

Supplementary Figure S3. Comparison of HAI (log2) titers between COV-Flu and mono-Flu participants vaccinated with Flucelvax and/or Flumist influenza vaccine in adults (18-64). The titers shown against influenza A (IAV/H1N1) (*A*) and IAV/H3N2) (*B*) and influenza B (IBV/Yamagata) (*C*) and IBV/Victoria) (*D*) virus components in COV-Flu and mono-Flu vaccinated participants. Numbers of participants in Flucelvax COV-Flu, n=11; in mono-Flu, n=9. Numbers of participants vaccinated with Flumist in COV-Flu, n=6; and in mono-Flu, n=6. The dotted line represents the seropositivity limit of log2 40 HAI. A non-parametric Mann-Whitney test have been performed comparing two groups (*p<0.05, ns: not significant).

Supplementary Table S1. The numbers and percentages of participants by vaccine type across three consecutive seasons included in the study groups.

Vaccine		2021-2022 season					
		COV-Flu[a] study group (n=109)		Mono-Flu[b] study group (n=56)		Mono-COVID-19[c] study group (n=39)	
		Young-adult (18-64 y.o.[d])	Elder (65-90 y.o.)	Young-adult (18-64 y.o.)	Elder (65-90 y.o.)	Young-adult (18-64 y.o.)	Elder (65-90 y.o.)
COVID-19	Moderna (mRNA-1273)	17 (26%)	4 (9%)	-	-	5 (21%)	6 (40%)
	Pfizer (BNT162b2)	49 (74%)	39 (91%)	-	-	19 (79%)	9 (60%)
Influenza	Fluzone SD[e]-QIV[f]	41 (62%)	1 (2%)	26 (63%)	-	-	-
	FluBlok-QIV	25 (38%)	7 (16%)	15 (37%)	-	-	-
	Fluzone HD[g]-QIV	-	35 (82%)	-	15 (100%)	-	-

Vaccine		2022-2023 season					
		COV-Flu study group (n=61)		Mono-Flu study group (n=48)		Mono-COVID-19 study group (n=27)	
		Young-adult (18-64 y.o.)	Elder (65-90 y.o.)	Young-adult (18-64 y.o.)	Elder (65-90 y.o.)	Young-adult (18-64 y.o.)	Elder (65-90 y.o.)
COVID-19	Moderna (mRNA-1273)	5 (17%)	7 (23%)	-	-	6 (40%)	5 (42%)
	Pfizer (BNT162b2)	25 (83%)	24 (77%)	-	-	9 (60%)	7 (58%)
Influenza	Fluzone SD-QIV	15 (50%)	-	11 (48%)	-	-	-
	FluBlok-QIV	7 (23%)	-	5 (22%)	-	-	-
	Fluzone HD-QIV	-	31 (100%)	-	25 (100%)	-	-
	FluMist- QIV	3 (10%)	-	3 (13%)	-	-	-
	Flucelvax-QIV	5 (17%)	-	4 (17%)	-	-	-

Vaccine		2023-2024 season					
		COV-Flu study group (n=31)		Mono-Flu study group (n=26)		Mono-COVID-19 study group (n=1)	
		Young-adult (18-64 y.o.)	Elder (65-90 y.o.)	Young-adult (18-64 y.o.)	Elder (65-90 y.o.)	Young-adult (18-64 y.o.)	Elder (65-90 y.o.)
COVID-19	Moderna (mRNA-1273)	3 (17%)	4 (31%)	-	-	1 (100%)	-
	Pfizer (BNT162b2)	15 (83%)	8 (61%)	-	-	-	-
	Unknown	-	1 (8%)	-	-	-	-
Influenza	Fluzone SD-QIV	9 (50%)	-	8 (50%)	-	-	-
	FluAd-QIV	-	-	-	-	-	-
	Fluzone HD-QIV	-	13 (100%)	-	10 (100%)	-	-
	FluMist- QIV	3 (17%)	-	3 (19%)	-	-	-
	Flucelvax-QIV	6 (33%)	-	5 (31%)	-	-	-

[a]COV-Flu: COVID-19 and Flu vaccination within a three-month. [b]Mono-Flu: Influenza monovaccinated. [c]Mono-COVID-19: COVID-19 monovaccinated. [d]Y.o.: Years old. [e]SD: Standard dose. [f]QIV: Quadrivalent Influenza vaccine. [g]HD: High dose.

Supplementary Table S2. Hemagglutination inhibition (HAI) titers against various influenza virus strains in participants. The demographics of the participants vaccinated with both COVID-19 mRNA and Flu vaccines (COV-Flu) and monovaccinated with influenza (mono-Flu) are shown with age, sex, and the type of influenza vaccine they received. Sera samples were tested at Day 0 and Day 28 against four influenza vaccine components.

									HAI Titers[a]							
									IAV[b]-H1N1		IAV-H3N2		IBV[c]-Yamagata		IBV-Victoria	
**GROUP**	**UGA[d]**	**IDs[e]**	**Date FLU Vaccinated**	**Date COVID-19 mRNA Vaccinated**	**Age**	**Sex**	**Flu Vaccine**	**mRNA vaccine**	**D0[f]**	**D28[g]**	**D0**	**D28**	**D0**	**D28**	**D0**	**D28**
COV-Flu [h]	UGA6	57	11/30/2021	11/1/2021	20	F[i]	FB[j]	Pfizer	40	640	40	160	10	80	5	5
COV-Flu	UGA6	61	11/30/2021	11/28/2021	21	F	FB	Pfizer	10	320	20	1280	80	640	10	80
COV-Flu	UGA6	70	11/29/2021	11/27/2021	22	M[k]	FB	Moderna	40	160	40	160	80	160	80	160
COV-Flu	UGA6	66	12/3/2021	12/10/2021	23	M	FB	Pfizer	5	80	80	80	10	80	5	20
COV-Flu	UGA6	36	11/2/2021	10/15/2021	24	F	FB	Pfizer	20	640	20	80	320	640	20	160
COV-Flu	UGA6	41	11/8/2021	11/22/2021	24	M	FB	Moderna	40	640	80	640	640	1280	40	80
COV-Flu	UGA6	12	10/12/2021	9/28/2021	25	F	FB	Pfizer	20	80	20	320	80	160	5	5
COV-Flu	UGA6	72	12/6/2021	11/19/2021	25	F	FB	Pfizer	40	80	80	320	80	160	80	160
COV-Flu	UGA6	85	12/13/2021	10/16/2021	28	F	FB	Moderna	80	320	10	80	160	160	5	10
COV-Flu	UGA6	53	11/12/2021	10/11/2021	30	F	FB	Pfizer	40	320	40	640	80	320	5	40
COV-Flu	UGA6	55	11/12/2021	10/27/2021	30	M	FB	Moderna	20	320	5	80	40	320	5	80
COV-Flu	UGA6	78	12/7/2021	11/21/2021	31	F	FB	Pfizer	5	80	5	5	10	160	5	40
COV-Flu	UGA6	86	12/10/2021	11/27/2021	33	F	FB	Pfizer	40	160	10	320	80	80	40	40
COV-Flu	UGA6	13	10/19/2021	11/11/2021	34	F	FB	Pfizer	40	80	40	160	160	320	5	20
COV-Flu	UGA6	68	12/7/2021	11/21/2021	35	M	FB	Pfizer	40	1280	40	640	160	640	20	40
COV-Flu	UGA6	51	11/29/2021	12/22/2021	36	M	FB	Pfizer	40	320	160	160	1280	640	5	10
COV-Flu	UGA6	44	11/2/2021	9/27/2021	40	F	FB	Pfizer	20	80	80	320	640	640	40	80
COV-Flu	UGA6	77	12/3/2021	12/17/2021	53	M	FB	Pfizer	20	320	20	80	160	320	80	160
COV-Flu	UGA6	69	11/30/2021	11/2/2021	55	F	FB	Pfizer	20	160	5	40	10	20	5	40
COV-Flu	UGA6	75	12/6/2021	11/28/2021	55	M	FB	Pfizer	5	80	5	40	40	640	10	80
COV-Flu	UGA6	67	11/29/2021	11/17/2021	59	F	FB	Moderna	10	20	10	10	160	160	80	80
COV-Flu	UGA6	60	11/29/2021	11/21/2021	60	M	FB	Pfizer	20	20	10	40	80	40	40	40
COV-Flu	UGA6	71	11/30/2021	10/30/2021	60	F	FB	Pfizer	10	160	5	40	40	160	20	40
COV-Flu	UGA6	28	10/25/2021	10/26/2021	62	F	FB	Pfizer	20	160	160	160	80	320	10	40

COV-Flu	UGA6	31	10/26/2021	11/1/2021	63	M	FB	Moderna	40	320	5	80	20	80	5	10
COV-Flu	UGA6	45	11/3/2021	9/28/2021	66	F	FB	Pfizer	10	80	5	40	40	160	40	80
COV-Flu	UGA6	26	10/25/2021	9/28/2021	68	F	FB	Pfizer	40	640	320	640	80	320	10	80
COV-Flu	UGA6	11	10/12/2021	10/7/2021	73	F	FB	Pfizer	20	80	10	20	320	640	10	10
COV-Flu	UGA6	37	11/1/2021	9/30/2021	73	M	FB	Pfizer	20	40	40	320	20	80	80	80
COV-Flu	UGA6	43	11/3/2021	10/14/2021	74	F	FB	Pfizer	5	10	20	80	20	80	40	320
COV-Flu	UGA6	40	11/8/2021	11/19/2021	79	F	FB	Pfizer	10	640	5	80	40	320	160	320
COV-Flu	UGA6	48	11/9/2021	10/20/2021	83	F	FB	Pfizer	40	40	40	160	160	160	80	160
COV-Flu	UGA6	FB1-087	12/14/2021	-	20	F	FB	-	80	320	20	160	80	160	80	160
COV-Flu	UGA6	FB1-073	12/7/2021	-	21	F	FB	-	10	320	40	320	80	80	5	20
COV-Flu	UGA6	FB1-004	9/15/2021	-	24	M	FB	-	20	320	80	80	80	160	80	80
COV-Flu	UGA6	FB1-030	11/1/2021	-	24	F	FB	-	40	640	20	160	40	80	20	80
COV-Flu	UGA6	FB1-006	10/5/2021	-	25	F	FB	-	80	320	160	640	160	320	40	40
COV-Flu	UGA6	FB1-002	9/28/2021	-	27	F	FB	-	80	320	320	640	160	320	160	320
COV-Flu	UGA6	FB1-016	10/15/2021	-	29	M	FB	-	10	160	40	320	40	80	5	5
COV-Flu	UGA6	FB1-003	9/15/2021	-	37	M	FB	-	40	320	80	80	80	160	20	40
COV-Flu	UGA6	FB1-050	11/9/2021	-	37	F	FB	-	20	80	80	160	320	640	20	40
COV-Flu	UGA6	FB1-063	11/29/2021	-	37	F	FB	-	10	20	5	80	20	80	10	20
COV-Flu	UGA6	FB1-023	10/26/2021	-	38	F	FB	-	40	640	80	160	160	160	80	80
COV-Flu	UGA6	FB1-047	11/5/2021	-	57	M	FB	-	10	160	40	40	160	320	40	80
COV-Flu	UGA6	FB1-015	10/18/2021	-	62	F	FB	-	160	320	80	640	160	320	20	160
COV-Flu	UGA6	FB1-035	11/2/2021	-	63	F	FB	-	10	40	20	80	80	80	20	40
COV-Flu	UGA6	FB1-054	11/12/2021	-	63	M	FB	-	40	80	5	160	10	80	10	40
COV-Flu	UGA7	FB2-044	10/19/2022	-	41	F	FB	-	20	20	80	40	320	640	10	5
COV-Flu	UGA7	FB2-082	11/3/2022	-	41	F	FB	-	80	80	10	20	40	80	5	5
COV-Flu	UGA7	FB2-110	11/8/2022	-	41	M	FB	-	80	80	10	160	10	5	40	5
COV-Flu	UGA7	FB2-103	10/31/2022	-	50	F	FB	-	10	20	20	40	80	80	10	5
COV-Flu	UGA7	FB2-033	10/28/2022	-	58	M	FB	-	160	40	10	10	10	5	5	5
COV-Flu	UGA7	FB2-036	11/8/2022	10/24/2022	25	F	FB	Pfizer	80	80	20	80	160	320	10	5
COV-Flu	UGA7	FB2-055	11/11/2022	10/9/2022	31	M	FB	Pfizer	320	320	10	80	160	320	5	5
COV-Flu	UGA7	FB2-062	11/11/2022	10/22/2022	41	M	FB	Pfizer	40	40	10	320	20	40	5	5
COV-Flu	UGA7	FB2-094	10/13/2022	9/9/2022	44	M	FB	Pfizer	80	160	10	320	20	1280	10	160
COV-Flu	UGA7	FB2-095	10/19/2022	10/18/2022	48	F	FB	Pfizer	20	20	5	5	10	80	5	10
COV-Flu	UGA7	FB2-099	10/31/2022	11/3/2022	56	M	FB	Pfizer	20	160	5	40	320	640	80	5
COV-Flu	UGA7	FB2-111	11/9/2022	10/3/2022	59	F	FB	Moderna	10	40	20	40	320	1280	80	320

COV-Flu	UGA6	379	10/4/2021	10/2/2021	65	F	FZ-HD[m]	Pfizer	10	160	10	10	10	10	5	5
COV-Flu	UGA6	14	9/21/2021	10/6/2021	66	F	FZ-HD	Pfizer	5	40	10	20	5	20	5	20
COV-Flu	UGA6	278	10/7/2021	9/28/2021	66	M	FZ-HD	Pfizer	10	80	40	80	10	20	20	20
COV-Flu	UGA6	47	10/26/2021	10/18/2021	67	F	FZ-HD	Pfizer	20	20	20	20	20	20	20	20
COV-Flu	UGA6	270	10/5/2021	9/29/2021	67	M	FZ-HD	Pfizer	80	80	40	160	160	160	20	80
COV-Flu	UGA6	304	9/28/2021	8/19/2021	67	F	FZ-HD	Moderna	20	40	80	80	40	320	40	80
COV-Flu	UGA6	369	9/24/2021	10/2/2021	67	M	FZ-HD	Pfizer	20	40	20	160	160	160	40	40
COV-Flu	UGA6	295	9/22/2021	9/27/2021	68	F	FZ-HD	Pfizer	80	160	10	20	20	40	40	80
COV-Flu	UGA6	296	9/22/2021	9/27/2021	68	M	FZ-HD	Pfizer	5	80	5	20	10	20	20	20
COV-Flu	UGA6	126	9/20/2021	7/30/2021	69	M	FZ-HD	Pfizer	5	80	20	40	20	40	20	20
COV-Flu	UGA6	334	10/11/2021	9/28/2021	69	M	FZ-HD	Pfizer	80	160	5	20	80	80	40	80
COV-Flu	UGA6	335	10/11/2021	9/28/2021	69	F	FZ-HD	Pfizer	20	160	5	160	40	40	10	40
COV-Flu	UGA6	481	10/19/2021	10/29/2021	69	F	FZ-HD	Moderna	5	40	20	320	20	160	20	80
COV-Flu	UGA6	149	11/9/2021	11/10/2021	70	M	FZ-HD	Moderna	10	40	40	40	5	10	5	20
COV-Flu	UGA6	276	9/30/2021	10/3/2021	70	F	FZ-HD	Pfizer	10	40	80	80	20	40	40	40
COV-Flu	UGA6	170	9/21/2021	9/7/2021	71	F	FZ-HD	Pfizer	10	20	40	80	5	20	10	20
COV-Flu	UGA6	294	10/19/2021	10/22/2021	71	F	FZ-HD	Pfizer	20	40	20	40	40	80	40	80
COV-Flu	UGA6	320	10/5/2021	10/20/2021	71	F	FZ-HD	Pfizer	5	10	10	40	10	20	10	20
COV-Flu	UGA6	345	10/14/2021	9/24/2021	72	F	FZ-HD	Pfizer	10	20	20	160	40	160	80	160
COV-Flu	UGA6	135	10/5/2021	9/8/2021	73	M	FZ-HD	Pfizer	20	20	10	20	20	20	80	80
COV-Flu	UGA6	148	9/20/2021	10/7/2021	73	M	FZ-HD	Pfizer	10	20	5	20	10	10	40	20
COV-Flu	UGA6	177	11/8/2021	11/15/2021	73	M	FZ-HD	Moderna	10	40	40	160	20	20	20	20
COV-Flu	UGA6	308	9/23/2021	10/2/2021	73	M	FZ-HD	Pfizer	10	10	5	10	160	160	20	40
COV-Flu	UGA6	326	10/5/2021	9/26/2021	73	M	FZ-HD	Pfizer	10	20	80	80	20	40	20	20
COV-Flu	UGA6	273	9/22/2021	9/27/2021	74	M	FZ-HD	Pfizer	10	10	5	20	20	20	40	40
COV-Flu	UGA6	274	9/22/2021	9/27/2021	74	F	FZ-HD	Pfizer	10	20	5	20	5	10	20	20
COV-Flu	UGA6	353	10/21/2021	10/29/2021	74	M	FZ-HD	Pfizer	10	40	5	20	5	10	40	40
COV-Flu	UGA6	433	9/29/2021	9/25/2021	75	M	FZ-HD	Pfizer	40	80	160	320	20	40	80	80
COV-Flu	UGA6	162	9/20/2021	9/27/2021	77	F	FZ-HD	Pfizer	20	40	80	80	5	20	40	80
COV-Flu	UGA6	362	9/21/2021	10/4/2021	77	M	FZ-HD	Pfizer	10	20	320	320	20	40	40	80
COV-Flu	UGA6	169	9/21/2021	9/28/2021	78	M	FZ-HD	Pfizer	10	5	10	10	10	20	20	40
COV-Flu	UGA6	291	10/14/2021	9/18/2021	79	F	FZ-HD	Pfizer	5	40	20	20	40	80	40	40
COV-Flu	UGA6	376	10/14/2021	10/11/2021	80	M	FZ-HD	Pfizer	10	80	80	320	20	20	40	40
COV-Flu	UGA6	298	9/23/2021	9/1/2021	82	F	FZ-HD	Pfizer	10	40	40	80	20	40	10	40
COV-Flu	UGA6	455	9/24/2021	10/14/2021	86	F	FZ-HD	Pfizer	5	160	320	320	40	320	40	320

COV-Flu	UGA7	565	10/24/2022	9/8/2022	65	F	FZ-HD	Pfizer	20	160	5	80	160	640	5	20
COV-Flu	UGA7	309	9/27/2022	9/15/2022	66	F	FZ-HD	Moderna	20	320	5	1280	20	160	40	160
COV-Flu	UGA7	354	10/24/2022	9/30/2022	66	M	FZ-HD	Pfizer	5	20	5	20	5	10	640	1280
COV-Flu	UGA7	379	11/4/2022	11/7/2022	66	F	FZ-HD	Pfizer	20	80	5	5	10	5	5	5
COV-Flu	UGA7	611	10/25/2022	9/20/2022	66	M	FZ-HD	Pfizer	40	80	5	5	80	160	80	640
COV-Flu	UGA7	380	11/4/2022	11/7/2022	67	M	FZ-HD	Pfizer	10	40	5	5	5	20	80	160
COV-Flu	UGA7	323	10/19/2022	10/2/2022	68	F	FZ-HD	Pfizer	40	160	5	160	10	20	40	5
COV-Flu	UGA7	369	10/31/2022	10/28/2022	68	M	FZ-HD	Pfizer	20	40	5	10	320	640	20	5
COV-Flu	UGA7	566	10/24/2022	9/8/2022	68	M	FZ-HD	Pfizer	5	10	10	20	160	320	160	320
COV-Flu	UGA7	632	11/4/2022	9/28/2022	68	F	FZ-HD	Moderna	40	80	5	20	20	320	20	160
COV-Flu	UGA7	196	9/27/2022	9/15/2022	69	M	FZ-HD	Moderna	10	20	10	40	10	40	40	80
COV-Flu	UGA7	304	11/7/2022	10/13/2022	69	F	FZ-HD	Pfizer	10	40	10	640	80	320	20	5
COV-Flu	UGA7	588	10/31/2022	10/3/2022	69	F	FZ-HD	Pfizer	160	1280	160	640	80	320	20	40
COV-Flu	UGA7	612	10/12/2022	10/10/2022	69	M	FZ-HD	Pfizer	20	80	80	640	5	160	20	640
COV-Flu	UGA7	126	9/30/2022	10/2/2022	70	M	FZ-HD	Moderna	20	80	10	80	20	320	20	80
COV-Flu	UGA7	568	9/28/2022	10/23/2022	70	F	FZ-HD	Pfizer	40	80	5	5	40	80	10	40
COV-Flu	UGA7	583	10/14/2022	10/9/2022	71	M	FZ-HD	Pfizer	5	5	10	10	20	20	20	40
COV-Flu	UGA7	98	10/5/2022	10/25/2022	72	M	FZ-HD	Pfizer	40	40	5	5	80	160	320	320
COV-Flu	UGA7	320	10/13/2022	9/29/2022	72	F	FZ-HD	Pfizer	5	10	5	20	5	40	5	80
COV-Flu	UGA7	148	10/19/2022	10/17/2022	74	M	FZ-HD	Pfizer	10	40	20	5	10	20	20	40
COV-Flu	UGA7	177	9/27/2022	9/8/2022	74	M	FZ-HD	Pfizer	40	40	5	40	20	40	20	80
COV-Flu	UGA7	593	10/11/2022	9/12/2022	74	F	FZ-HD	Pfizer	20	40	5	10	320	640	5	20
COV-Flu	UGA7	381	11/9/2022	9/19/2022	75	F	FZ-HD	Moderna	10	40	5	160	10	40	5	5
COV-Flu	UGA7	594	10/11/2022	10/1/2022	75	M	FZ-HD	Pfizer	40	80	5	40	5	5	10	20
COV-Flu	UGA7	637	10/19/2022	9/10/2022	75	F	FZ-HD	Pfizer	10	20	20	80	80	160	5	5
COV-Flu	UGA7	615	10/12/2022	9/8/2022	76	M	FZ-HD	Pfizer	20	40	20	40	20	40	20	40
COV-Flu	UGA7	362	11/9/2022	11/11/2022	78	M	FZ-HD	Pfizer	10	10	20	40	40	20	40	40
COV-Flu	UGA7	169	9/28/2022	9/25/2022	79	M	FZ-HD	Moderna	20	20	5	10	20	40	20	20
COV-Flu	UGA7	651	11/2/2022	11/2/2022	80	F	FZ-HD	Pfizer	320	640	5	160	40	80	160	160
COV-Flu	UGA7	376	10/25/2022	10/24/2022	81	M	FZ-HD	Moderna	10	20	10	40	20	40	20	40
COV-Flu	UGA7	455	10/19/2022	10/14/2022	87	F	FZ-HD	Pfizer	40	160	80	2560	80	1280	80	320
COV-Flu	UGA8	306	9/18/2023	9/25/2023	65	F	FZ-HD	UNKNOWN	5	20	5	10	160	160	160	320
COV-Flu	UGA8	667	9/19/2023	10/10/2023	67	M	FZ-HD	Pfizer	80	5	5	5	160	80	40	160
COV-Flu	UGA8	323	9/27/2023	10/4/2023	69	F	FZ-HD	Moderna	40	80	20	40	20	80	40	80
COV-Flu	UGA8	633	9/27/2023	10/1/2023	69	M	FZ-HD	Moderna	160	160	160	160	160	320	320	640

COV-Flu	UGA8	304	10/11/2023	9/27/2023	69	F	FZ-HD	Moderna	5	5	160	320	160	1280	40	160
COV-Flu	UGA8	295	9/20/2023	10/2/2023	70	F	FZ-HD	Pfizer	10	5	5	20	80	80	5120	1280
COV-Flu	UGA8	568	9/19/2023	9/29/2023	71	F	FZ-HD	Pfizer	40	5	40	80	320	160	80	40
COV-Flu	UGA8	276	9/19/2023	9/28/2023	72	F	FZ-HD	Pfizer	5	5	40	40	80	80	80	80
COV-Flu	UGA8	170	9/12/2023	10/2/2023	73	F	FZ-HD	Pfizer	5	10	20	80	80	80	20	40
COV-Flu	UGA8	618	9/13/2023	9/16/2023	74	M	FZ-HD	Pfizer	5	10	5	5	160	160	320	160
COV-Flu	UGA8	353	9/22/2023	10/18/2023	76	M	FZ-HD	Pfizer	5	5	40	80	80	5	5	40
COV-Flu	UGA8	640	9/19/2023	10/5/2023	77	F	FZ-HD	Moderna	5	5	20	20	10	40	80	160
COV-Flu	UGA8	169	9/12/2023	10/2/2023	80	M	FZ-HD	Pfizer	5	5	10	5	80	40	40	40
Mono-Flu	UGA6	535	10/12/2021	-	65	F	FZ-HD	-	40	160	20	160	40	160	20	40
Mono-Flu	UGA6	293	9/28/2021	-	66	F	FZ-HD	-	20	40	80	160	40	40	20	40
Mono-Flu	UGA6	391	9/29/2021	-	66	F	FZ-HD	-	20	160	10	20	20	40	10	20
Mono-Flu	UGA6	283	9/29/2021	-	67	F	FZ-HD	-	20	20	20	40	20	40	20	40
Mono-Flu	UGA6	268	9/24/2021	-	69	F	FZ-HD	-	5	80	20	80	5	10	10	20
Mono-Flu	UGA6	184	9/27/2021	-	70	M	FZ-HD	-	10	20	5	5	5	20	20	20
Mono-Flu	UGA6	319	10/27/2021	-	70	F	FZ-HD	-	5	40	40	40	10	20	10	20
Mono-Flu	UGA6	185	9/21/2021	-	71	F	FZ-HD	-	10	40	10	20	10	10	10	20
Mono-Flu	UGA6	332	9/27/2021	-	71	F	FZ-HD	-	5	5	5	10	5	5	5	10
Mono-Flu	UGA6	437	10/6/2021	-	71	F	FZ-HD	-	10	40	20	80	20	40	10	20
Mono-Flu	UGA6	125	10/14/2021	-	72	F	FZ-HD	-	5	160	10	80	5	20	5	40
Mono-Flu	UGA6	134	9/23/2021	-	74	F	FZ-HD	-	10	10	20	40	20	20	10	20
Mono-Flu	UGA6	269	9/27/2021	-	75	F	FZ-HD	-	20	20	5	40	5	10	40	80
Mono-Flu	UGA6	209	10/4/2021	-	80	F	FZ-HD	-	10	40	10	10	10	10	40	40
Mono-Flu	UGA6	171	9/24/2021	-	83	F	FZ-HD	-	10	20	20	80	20	40	80	80
Mono-Flu	UGA7	595	10/28/2022	-	66	M	FZ-HD	-	5	80	10	80	10	160	5	5
Mono-Flu	UGA7	14	10/20/2022	-	67	F	FZ-HD	-	5	20	5	40	5	5	5	5
Mono-Flu	UGA7	203	10/11/2022	-	67	M	FZ-HD	-	20	40	5	20	40	80	20	160
Mono-Flu	UGA7	205	10/3/2022	-	67	F	FZ-HD	-	40	40	5	5	5	40	5	10
Mono-Flu	UGA7	278	11/2/2022	-	67	M	FZ-HD	-	5	5	5	5	5	5	5	5
Mono-Flu	UGA7	582	10/4/2022	-	68	F	FZ-HD	-	20	80	5	40	10	160	40	80
Mono-Flu	UGA7	589	10/4/2022	-	68	F	FZ-HD	-	20	20	5	10	20	80	5	20
Mono-Flu	UGA7	584	10/14/2022	-	69	F	FZ-HD	-	20	160	5	40	10	40	40	160
Mono-Flu	UGA7	77	9/27/2022	-	70	F	FZ-HD	-	20	40	5	5	20	40	10	80
Mono-Flu	UGA7	335	10/20/2022	-	70	F	FZ-HD	-	40	80	5	40	10	40	20	40
Mono-Flu	UGA7	476	11/10/2022	-	70	M	FZ-HD	-	5	10	5	20	160	320	5	5

Mono-Flu	UGA7	276	10/31/2022	-	71	F	FZ-HD	-	10	20	40	80	20	160	20	80
Mono-Flu	UGA7	314	11/2/2022	-	71	F	FZ-HD	-	20	20	5	5	5	10	40	80
Mono-Flu	UGA7	83	10/13/2022	-	72	F	FZ-HD	-	5	10	5	20	5	5	5	5
Mono-Flu	UGA7	286	10/6/2022	-	73	M	FZ-HD	-	20	20	5	5	10	10	10	10
Mono-Flu	UGA7	567	10/3/2022	-	73	M	FZ-HD	-	20	20	5	10	10	20	40	80
Mono-Flu	UGA7	618	10/27/2022	-	73	M	FZ-HD	-	5	10	5	5	80	160	80	160
Mono-Flu	UGA7	135	10/4/2022	-	74	M	FZ-HD	-	20	20	5	40	20	80	20	40
Mono-Flu	UGA7	273	10/13/2022	-	75	M	FZ-HD	-	5	10	5	40	10	20	20	40
Mono-Flu	UGA7	284	10/12/2022	-	75	M	FZ-HD	-	10	20	5	20	20	80	40	80
Mono-Flu	UGA7	308	10/18/2022	-	75	M	FZ-HD	-	5	20	5	10	160	20	10	20
Mono-Flu	UGA7	640	10/20/2022	-	76	F	FZ-HD	-	5	5	10	20	5	5	5	80
Mono-Flu	UGA7	569	9/29/2022	-	78	F	FZ-HD	-	10	20	5	5	5	10	10	20
Mono-Flu	UGA7	644	10/24/2022	-	81	F	FZ-HD	-	20	20	5	5	5	5	80	5
Mono-Flu	UGA7	643	10/24/2022	-	84	M	FZ-HD	-	40	40	10	10	5	5	20	20
Mono-Flu	UGA8	673	9/11/2023	-	65	M	FZ-HD	-	5	5	5	10	20	160	5	20
Mono-Flu	UGA8	14	9/21/2023	-	68	F	FZ-HD	-	5	5	20	20	80	20	10	80
Mono-Flu	UGA8	179	10/3/2023	-	69	F	FZ-HD	-	5	5	20	80	160	320	20	80
Mono-Flu	UGA8	632	9/27/2023	-	69	F	FZ-HD	-	5	5	5	20	80	160	40	160
Mono-Flu	UGA8	674	9/12/2023	-	70	F	FZ-HD	-	5	10	20	40	40	80	80	160
Mono-Flu	UGA8	335	9/26/2023	-	71	F	FZ-HD	-	5	5	5	20	40	80	40	40
Mono-Flu	UGA8	648	9/21/2023	-	73	F	FZ-HD	-	5	5	20	20	160	320	40	80
Mono-Flu	UGA8	567	9/11/2023	-	74	M	FZ-HD	-	5	5	5	5	80	80	160	80
Mono-Flu	UGA8	638	9/11/2023	-	76	F	FZ-HD	-	5	5	20	40	320	320	40	40
Mono-Flu	UGA8	639	9/11/2023	-	76	M	FZ-HD	-	5	5	5	20	40	80	40	40
COV-Flu	UGA6	563	11/10/2021	10/6/2021	18	F	FZ-SD[n]	Pfizer	20	40	320	320	20	20	40	40
COV-Flu	UGA6	550	9/30/2021	9/13/2021	19	F	FZ-SD	Moderna	40	320	160	160	80	160	40	160
COV-Flu	UGA6	85	12/6/2021	11/12/2021	22	F	FZ-SD	Pfizer	20	80	80	80	20	40	20	40
COV-Flu	UGA6	439	11/2/2021	11/10/2021	23	F	FZ-SD	Pfizer	20	20	80	80	20	40	20	40
COV-Flu	UGA6	459	12/13/2021	12/22/2021	24	F	FZ-SD	Pfizer	20	320	160	320	320	640	160	320
COV-Flu	UGA6	442	10/8/2021	9/28/2021	25	F	FZ-SD	Pfizer	10	160	80	80	40	80	10	40
COV-Flu	UGA6	65	10/7/2021	10/27/2021	28	F	FZ-SD	Moderna	40	80	80	80	160	320	20	40
COV-Flu	UGA6	5	10/18/2021	8/30/2021	29	F	FZ-SD	Pfizer	5	20	160	160	40	40	5	20
COV-Flu	UGA6	69	12/2/2021	10/27/2021	29	M	FZ-SD	Pfizer	40	40	40	80	80	160	10	40
COV-Flu	UGA6	232	10/18/2021	10/13/2021	29	F	FZ-SD	Pfizer	20	80	20	40	40	40	5	10
COV-Flu	UGA6	463	10/22/2021	11/5/2021	30	F	FZ-SD	Moderna	10	160	80	160	160	160	80	80

COV-Flu	UGA6	473	10/29/2021	11/12/2021	30	F	FZ-SD	Moderna	160	320	320	320	80	160	20	40
COV-Flu	UGA6	443	10/25/2021	10/21/2021	32	F	FZ-SD	Pfizer	10	40	40	80	160	640	20	80
COV-Flu	UGA6	1	10/4/2021	10/6/2021	33	F	FZ-SD	Pfizer	20	640	20	320	160	320	5	80
COV-Flu	UGA6	250	10/12/2021	10/26/2021	36	F	FZ-SD	Pfizer	80	80	20	80	160	160	20	20
COV-Flu	UGA6	491	10/12/2021	10/7/2021	38	M	FZ-SD	Pfizer	10	160	20	20	20	40	20	40
COV-Flu	UGA6	351	9/28/2021	10/4/2021	40	F	FZ-SD	Pfizer	10	80	5	160	10	40	20	40
COV-Flu	UGA6	486	12/8/2021	10/10/2021	40	F	FZ-SD	Pfizer	40	160	160	320	40	160	80	160
COV-Flu	UGA6	411	12/14/2021	11/13/2021	41	M	FZ-SD	Moderna	40	160	20	20	20	40	20	40
COV-Flu	UGA6	279	11/10/2021	10/22/2021	42	F	FZ-SD	Pfizer	40	320	40	80	5	20	20	40
COV-Flu	UGA6	434	11/4/2021	9/24/2021	42	F	FZ-SD	Moderna	20	40	20	80	40	80	160	320
COV-Flu	UGA6	479	10/29/2021	11/19/2021	42	F	FZ-SD	Pfizer	10	40	40	40	5	20	20	40
COV-Flu	UGA6	407	11/8/2021	11/22/2021	43	M	FZ-SD	Pfizer	20	80	40	40	20	40	20	40
COV-Flu	UGA6	260	10/25/2021	9/24/2021	44	F	FZ-SD	Pfizer	20	80	40	160	20	40	20	40
COV-Flu	UGA6	410	11/3/2021	11/17/2021	44	F	FZ-SD	Moderna	5	20	5	80	20	20	40	80
COV-Flu	UGA6	311	9/30/2021	9/27/2021	45	F	FZ-SD	Pfizer	5	20	10	20	10	10	20	20
COV-Flu	UGA6	355	10/19/2021	11/9/2021	45	F	FZ-SD	Pfizer	20	40	10	40	40	40	40	40
COV-Flu	UGA6	386	11/1/2021	9/24/2021	46	F	FZ-SD	Pfizer	10	160	160	320	20	40	80	160
COV-Flu	UGA6	457	10/6/2021	10/7/2021	46	M	FZ-SD	Pfizer	10	160	80	160	40	160	10	40
COV-Flu	UGA6	328	10/28/2021	11/22/2021	48	F	FZ-SD	Moderna	20	160	20	80	20	20	20	80
COV-Flu	UGA6	377	12/7/2021	11/8/2021	49	F	FZ-SD	Pfizer	20	160	5	320	20	80	20	80
COV-Flu	UGA6	246	9/23/2021	10/8/2021	50	F	FZ-SD	Pfizer	10	80	10	320	20	40	40	160
COV-Flu	UGA6	392	9/30/2021	10/5/2021	50	F	FZ-SD	Pfizer	40	160	10	160	40	80	80	80
COV-Flu	UGA6	8	9/22/2021	9/7/2021	51	F	FZ-SD	Pfizer	10	80	80	160	20	20	40	80
COV-Flu	UGA6	100	9/20/2021	10/6/2021	51	F	FZ-SD	Pfizer	10	10	10	20	10	10	40	40
COV-Flu	UGA6	468	11/4/2021	9/9/2021	51	M	FZ-SD	Moderna	10	40	40	40	20	40	80	80
COV-Flu	UGA6	321	10/8/2021	10/27/2021	52	F	FZ-SD	Moderna	5	10	5	40	10	10	10	20
COV-Flu	UGA6	359	10/14/2021	10/27/2021	59	F	FZ-SD	Moderna	20	20	80	40	40	40	10	20
COV-Flu	UGA6	352	10/13/2021	11/5/2021	60	M	FZ-SD	Pfizer	40	320	40	160	40	80	40	160
COV-Flu	UGA6	384	10/27/2021	11/13/2021	60	F	FZ-SD	Pfizer	5	80	80	160	20	40	10	20
COV-Flu	UGA6	333	10/22/2021	11/10/2021	63	F	FZ-SD	Pfizer	10	160	5	80	40	160	40	80
COV-Flu	UGA6	354	11/12/2021	10/8/2021	65	M	FZ-SD	Pfizer	5	40	80	80	5	10	160	320
COV-Flu	UGA7	634	10/26/2022	9/28/2022	18	F	FZ-SD	Pfizer	80	160	5	320	80	320	40	1280
COV-Flu	UGA7	628	10/26/2022	9/28/2022	39	F	FZ-SD	Pfizer	5	320	5	160	10	320	5	80
COV-Flu	UGA7	322	10/26/2022	10/4/2022	46	F	FZ-SD	Pfizer	20	80	10	40	80	160	40	5
COV-Flu	UGA7	387	11/11/2022	12/6/2022	46	M	FZ-SD	Moderna	20	20	10	10	160	320	80	160

COV-Flu	UGA7	620	11/1/2022	11/23/2022	49	M	FZ-SD	Pfizer	20	40	10	40	320	320	80	640
COV-Flu	UGA7	392	11/2/2022	9/19/2022	51	F	FZ-SD	Pfizer	40	160	20	640	40	640	5	640
COV-Flu	UGA7	100	10/11/2022	9/22/2022	52	F	FZ-SD	Moderna	10	20	5	40	5	40	5	20
COV-Flu	UGA7	321	10/17/2022	9/3/2022	53	F	FZ-SD	Pfizer	5	10	5	5	5	5	5	5
COV-Flu	UGA7	340	10/31/2022	10/20/2022	53	M	FZ-SD	Pfizer	10	10	5	40	80	160	40	5
COV-Flu	UGA7	417	11/2/2022	10/4/2022	55	M	FZ-SD	Pfizer	5	5	5	5	10	5	20	5
COV-Flu	UGA7	416	11/9/2022	9/21/2022	59	F	FZ-SD	Pfizer	5	10	5	20	20	80	80	80
COV-Flu	UGA7	384	11/9/2022	9/20/2022	61	F	FZ-SD	Pfizer	10	20	10	640	20	80	5	10
COV-Flu	UGA7	599	10/13/2022	9/7/2022	62	M	FZ-SD	Pfizer	20	320	5	20	40	160	5	40
COV-Flu	UGA7	613	10/12/2022	10/10/2022	62	F	FZ-SD	Pfizer	80	80	5	10	20	40	40	80
COV-Flu	UGA7	306	10/25/2022	9/20/2022	63	F	FZ-SD	Moderna	40	80	5	5	80	160	40	80
COV-Flu	UGA8	250	9/19/2023	8/2/2023	38	F	FZ-SD	Pfizer	40	5	5	10	640	320	10	20
COV-Flu	UGA8	662	9/13/2023	9/9/2023	47	F	FZ-SD	Pfizer	5	5	80	40	80	80	5	10
COV-Flu	UGA8	708	10/9/2023	10/28/2023	51	F	FZ-SD	Pfizer	5	5	5	20	320	640	320	320
COV-Flu	UGA8	682	9/13/2023	9/16/2023	59	F	FZ-SD	Pfizer	5	10	20	80	40	40	80	40
COV-Flu	UGA8	359	10/18/2023	11/2/2023	61	F	FZ-SD	Pfizer	5	5	5	20	40	160	20	80
COV-Flu	UGA8	352	9/25/2023	9/9/2023	62	M	FZ-SD	Pfizer	5	5	20	40	80	160	80	80
COV-Flu	UGA8	384	9/15/2023	10/9/2023	62	F	FZ-SD	Pfizer	5	10	320	160	320	160	20	40
COV-Flu	UGA8	602	9/29/2023	9/27/2023	62	F	FZ-SD	Pfizer	5	5	10	20	40	80	20	40
COV-Flu	UGA8	629	9/28/2023	10/21/2023	64	F	FZ-SD	Pfizer	5	5	40	80	80	80	160	160
Mono-Flu	UGA6	558	10/13/2021	-	18	F	FZ-SD	-	40	80	320	320	80	80	80	160
Mono-Flu	UGA6	426	10/21/2021	-	19	F	FZ-SD	-	40	40	160	160	40	40	40	80
Mono-Flu	UGA6	450	10/6/2021	-	25	F	FZ-SD	-	40	80	80	320	160	320	80	80
Mono-Flu	UGA6	211	10/25/2021	-	27	M	FZ-SD	-	80	160	40	80	40	40	10	20
Mono-Flu	UGA6	29	10/19/2021	-	28	F	FZ-SD	-	20	160	160	160	160	160	80	160
Mono-Flu	UGA6	161	10/29/2021	-	29	M	FZ-SD	-	160	160	160	160	160	160	40	40
Mono-Flu	UGA6	471	11/3/2021	-	29	F	FZ-SD	-	40	80	80	80	80	80	40	80
Mono-Flu	UGA6	497	11/2/2021	-	33	F	FZ-SD	-	40	40	20	20	40	40	20	20
Mono-Flu	UGA6	396	9/22/2021	-	36	F	FZ-SD	-	40	80	80	80	80	80	20	20
Mono-Flu	UGA6	207	10/13/2021	-	37	M	FZ-SD	-	20	40	20	40	80	80	40	80
Mono-Flu	UGA6	225	10/22/2021	-	37	M	FZ-SD	-	20	40	40	80	160	160	10	10
Mono-Flu	UGA6	128	11/3/2021	-	40	F	FZ-SD	-	40	80	80	160	20	40	20	40
Mono-Flu	UGA6	215	9/20/2021	-	40	F	FZ-SD	-	5	40	80	80	20	20	40	40
Mono-Flu	UGA6	330	10/29/2021	-	41	F	FZ-SD	-	5	10	20	80	10	20	10	40
Mono-Flu	UGA6	492	11/1/2021	-	44	M	FZ-SD	-	20	10	5	5	5	40	80	80

Mono-Flu	UGA6	420	10/21/2021	-	46	F	FZ-SD	-	5	40	80	80	640	640	640	640
Mono-Flu	UGA6	66	10/6/2021	-	47	M	FZ-SD	-	5	10	80	160	10	10	160	160
Mono-Flu	UGA6	331	10/7/2021	-	48	F	FZ-SD	-	5	10	80	80	40	40	10	10
Mono-Flu	UGA6	431	9/20/2021	-	49	F	FZ-SD	-	20	20	20	40	80	160	320	640
Mono-Flu	UGA6	404	10/20/2021	-	50	F	FZ-SD	-	10	10	5	80	80	160	80	80
Mono-Flu	UGA6	316	10/11/2021	-	51	M	FZ-SD	-	5	20	5	5	5	10	5	10
Mono-Flu	UGA6	62	10/4/2021	-	52	F	FZ-SD	-	20	40	80	80	10	20	20	40
Mono-Flu	UGA6	317	10/11/2021	-	56	F	FZ-SD	-	5	20	40	80	5	10	10	20
Mono-Flu	UGA6	318	10/13/2021	-	57	M	FZ-SD	-	20	40	5	20	10	20	40	80
Mono-Flu	UGA6	370	10/25/2021	-	60	M	FZ-SD	-	20	20	10	10	320	320	160	160
Mono-Flu	UGA6	157	10/22/2021	-	64	M	FZ-SD	-	5	10	40	40	10	20	40	80
Mono-Flu	UGA7	559	10/12/2022	-	19	F	FZ-SD	-	40	80	40	320	5	20	5	20
Mono-Flu	UGA7	311	10/13/2022	-	46	F	FZ-SD	-	5	5	5	5	5	40	5	5
Mono-Flu	UGA7	378	12/5/2022	-	51	F	FZ-SD	-	40	80	5	20	40	80	5	5
Mono-Flu	UGA7	8	9/29/2022	-	52	F	FZ-SD	-	20	40	5	20	20	40	10	40
Mono-Flu	UGA7	518	11/29/2022	-	52	M	FZ-SD	-	20	40	80	160	40	80	5	5
Mono-Flu	UGA7	373	10/20/2022	-	54	F	FZ-SD	-	20	40	5	10	40	80	80	5
Mono-Flu	UGA7	352	11/10/2022	-	61	M	FZ-SD	-	80	80	20	40	80	160	80	80
Mono-Flu	UGA7	370	11/3/2022	-	61	M	FZ-SD	-	20	20	10	20	160	320	640	640
Mono-Flu	UGA7	403	9/30/2022	-	61	F	FZ-SD	-	20	40	20	80	10	20	20	40
Mono-Flu	UGA7	425	11/8/2022	-	62	F	FZ-SD	-	40	80	40	80	160	320	40	40
Mono-Flu	UGA7	86	10/4/2022	-	63	M	FZ-SD	-	160	160	5	10	40	80	20	40
Mono-Flu	UGA8	751	11/8/2023	-	29	M	FZ-SD	-	5	5	5	20	40	80	5	40
Mono-Flu	UGA8	491	10/16/2023	-	40	M	FZ-SD	-	5	5	5	5	40	40	10	40
Mono-Flu	UGA8	715	10/19/2023	-	47	F	FZ-SD	-	5	5	5	10	80	80	40	80
Mono-Flu	UGA8	432	9/15/2023	-	59	M	FZ-SD	-	5	5	40	20	80	20	160	80
Mono-Flu	UGA8	606	10/3/2023	-	59	F	FZ-SD	-	5	5	5	10	20	40	40	80
Mono-Flu	UGA8	600	9/18/2023	-	61	M	FZ-SD	-	5	5	5	5	160	80	80	80
Mono-Flu	UGA8	663	10/17/2023	-	61	F	FZ-SD	-	5	5	5	10	40	40	5	40
Mono-Flu	UGA8	347	10/10/2023	-	64	M	FZ-SD	-	5	5	40	80	80	160	80	80
COV-Flu	UGA7	FM2-121	11/3/2022	10/22/2022	21	F	FM°	Pfizer	5	5	5	5	80	80	5	5
COV-Flu	UGA7	FM2-113	10/31/2022	9/13/2022	30	M	FM	Pfizer	10	20	5	5	40	40	5	5
COV-Flu	UGA7	FM2-050	11/10/2022	9/15/2022	35	M	FM	Moderna	10	40	5	5	20	20	5	5
COV-Flu	UGA8	FM3-112	10/2/2023	9/26/2023	25	F	FM	Pfizer	5	5	5	10	20	40	5	40
COV-Flu	UGA8	FM3-104	9/14/2023	10/2/2023	36	M	FM	Moderna	20	10	640	320	640	320	40	40

COV-Flu	UGA8	FM3-105	9/14/2023	10/4/2023	39	F	FM	Moderna	5	5	5	5	160	160	20	10
Mono-Flu	UGA7	FM2-110	11/2/2022	-	22	F	FM	-	20	20	20	10	80	80	10	5
Mono-Flu	UGA7	FM2-142	12/6/2022	-	26	M	FM	-	5	5	5	5	5	5	5	5
Mono-Flu	UGA7	FM2-056	11/1/2022	-	39	M	FM	-	20	20	5	5	40	80	10	10
Mono-Flu	UGA8	FM3-156	9/19/2023	-	25	F	FM	-	5	5	5	5	320	160	20	20
Mono-Flu	UGA8	FM3-150	9/14/2023	-	31	M	FM	-	10	5	40	40	160	160	20	5
Mono-Flu	UGA8	FM3-161	9/25/2023	-	42	F	FM	-	20	10	10	10	40	40	5	10
COV-Flu	UGA7	FC1-010	10/19/2022	9/21/2022	25	F	FC[p]	Pfizer	20	40	5	5	10	5	5	5
COV-Flu	UGA7	FC1-028	11/7/2022	11/11/2022	29	F	FC	Pfizer	5	640	5	40	5	640	10	40
COV-Flu	UGA7	FC1-026	10/31/2022	9/15/2022	31	F	FC	Pfizer	80	80	20	40	160	640	80	160
COV-Flu	UGA7	FC1-008	10/20/2022	10/6/2022	35	F	FC	Pfizer	80	160	40	80	640	640	320	160
COV-Flu	UGA7	FC1-042	12/5/2022	1/1/2023	42	F	FC	Pfizer	5	160	5	80	20	320	20	640
COV-Flu	UGA8	FC2-077	9/14/2023	10/4/2023	31	M	FC	Pfizer	20	5	320	160	320	320	20	40
COV-Flu	UGA8	FC2-089	9/18/2023	10/10/2023	36	F	FC	Pfizer	5	5	20	40	80	80	5	5
COV-Flu	UGA8	FC2-099	9/26/2023	9/21/2023	42	F	FC	Pfizer	5	5	40	80	640	640	10	20
COV-Flu	UGA8	FC2-068	9/8/2023	9/5/2023	45	M	FC	Moderna	5	10	40	20	40	1280	40	80
COV-Flu	UGA8	FC2-102	9/27/2023	9/29/2023	46	F	FC	Pfizer	5	5	5	5	640	1280	5	320
COV-Flu	UGA8	FC2-044	9/18/2023	9/24/2023	64	F	FC	Pfizer	5	5	10	40	20	20	20	20
Mono-Flu	UGA7	FC1-014	10/18/2022	-	30	F	FC	-	40	40	5	40	80	160	5	5
Mono-Flu	UGA7	FC1-005	10/4/2022	-	31	F	FC	-	20	80	5	40	320	640	10	40
Mono-Flu	UGA7	FC1-001	9/29/2022	-	35	M	FC	-	20	160	40	5	80	160	40	40
Mono-Flu	UGA7	FC1-007	10/4/2022	-	44	F	FC	-	5	5	5	5	40	40	20	20
Mono-Flu	UGA8	FC2-083	9/14/2023	-	34	F	FC	-	5	5	5	5	80	80	5	40
Mono-Flu	UGA8	FC2-001	9/27/2023	-	36	M	FC	-	20	5	5	5	80	80	80	80
Mono-Flu	UGA8	FC2-049	9/7/2023	-	42	M	FC	-	5	20	5	5	40	40	40	40
Mono-Flu	UGA8	FC2-098	9/25/2023	-	42	F	FC	-	5	5	40	40	40	5	40	160
Mono-Flu	UGA8	FC2-109	10/2/2023	-	46	F	FC	-	5	5	20	20	160	160	40	40

[a]HAI: Hemagglutinin inhibition. [b]IAV: Influenza A virus. [c]IBV: Influenza B virus. [d] UGA: University of Georgia study cohorts; UGA6: 2021–2022 season cohort, UGA7: 2022–2023 season cohort, UGA8: 2023–2024 season cohort. [e]IDs: Identifications. [f]D0: Day 0. [g]D28: Day28. [h]COV-Flu: Vaccination with both COVID-19 mRNA and influenza vaccines. [i]F: Female. [j]FB: FluBlok. [k]M: Male. [l]Mono-Flu: Influenza monovaccinated. [m]FZ-HD: Fluzone high dose. [n]FZ-SD: Fluzone standard dose. [o]FM: Flumist. [p]FC: Flucelvaccine.

Supplementary Table S3. Pseudovirus neutralization titers against the Wuhan Hu-1 variant of SARS-CoV-2. Titers were determined using serum samples collected from mono-COVID and COV-Flu participants within 120 days following COVID-19 vaccination.

Cohort	Age group	Cohort ID (UGA/SPARTA)[a]	Age	Sex	Season	Flu Vaccine	mRNA vaccine	COVID-19 Vaccine no	Day interval between COVID-19 and Flu vaccine dates	PsVN IC50 RLU against Wuhan Hu-1[b]
COV-Flu[c]	18-64 Adult	UGA6-439/CTR-230	23	F[d]	2021-2022	FZ-SD[e]	3rd Vac	Pfizer	8	6575.08701
COV-Flu	18-64 Adult	UGA6-459	24	F	2021-2022	FZ-SD	3rd Vac	Pfizer	9	1653.00903
COV-Flu	18-64 Adult	UGA6-463	30	F	2021-2022	FZ-SD	3rd Vac	Moderna	14	998.31599
COV-Flu	18-64 Adult	UGA6-473	30	F	2021-2022	FZ-SD	3rd Vac	Moderna	14	470.09608
COV-Flu	18-64 Adult	UGA6-443/CTR-082	32	F	2021-2022	FZ-SD	3rd Vac	Pfizer	4	2447.44988
COV-Flu	18-64 Adult	UGA6-250	36	F	2021-2022	FZ-SD	3rd Vac	Pfizer	14	224.37784
COV-Flu	18-64 Adult	UGA6-351	40	F	2021-2022	FZ-SD	3rd Vac	Pfizer	6	854.56264
COV-Flu	18-64 Adult	UGA6-407	43	M[f]	2021-2022	FZ-SD	3rd Vac	Pfizer	14	6700.53966
COV-Flu	18-64 Adult	UGA6-457	46	M	2021-2022	FZ-SD	3rd Vac	Pfizer	1	483.90627
COV-Flu	18-64 Adult	UGA6-392/CTR-166	50	F	2021-2022	FZ-SD	3rd Vac	Pfizer	5	557.72122
COV-Flu	18-64 Adult	UGA6-359/CTR-159	59	F	2021-2022	FZ-SD	3rd Vac	Moderna	13	137.4391
COV-Flu	18-64 Adult	UGA6-384/CTR-031	60	F	2021-2022	FZ-SD	3rd Vac	Pfizer	17	563.2729
COV-Flu	18-64 Adult	UGA6-333/CTR-123	63	F	2021-2022	FZ-SD	3rd Vac	Pfizer	19	1427.17933
COV-Flu	18-64 Adult	UGA7-322	46	F	2022-2023	FZ-SD	Unknown	Pfizer	22	1110.87
COV-Flu	18-64 Adult	UGA7-387	46	M	2022-2023	FZ-SD	Unknown	Moderna	25	6461.07
COV-Flu	18-64 Adult	UGA7-620/CVI-956	49	M	2022-2023	FZ-SD	Unknown	Pfizer	22	275.5
COV-Flu	18-64 Adult	UGA7-392/CTR-166	51	F	2022-2023	FZ-SD	4th Vac	Pfizer	44	3039.12
COV-Flu	18-64 Adult	UGA7-100/CTR-044	52	F	2022-2023	FZ-SD	5th Vac	Moderna	19	2307.41
COV-Flu	18-64 Adult	UGA7-321/CVI-081	53	F	2022-2023	FZ-SD	Unknown	Pfizer	44	64.89
COV-Flu	18-64 Adult	UGA7-340/CTR-152	53	M	2022-2023	FZ-SD	Unknown	Pfizer	11	2180.32
COV-Flu	18-64 Adult	UGA7-417/CTR-221	55	M	2022-2023	FZ-SD	4th Vac	Pfizer	29	6824.55
COV-Flu	18-64 Adult	UGA7-416/CTR-220	59	F	2022-2023	FZ-SD	Unknown	Pfizer	49	4654.49

COV-Flu	18-64 Adult	UGA7-384/CTR-031	61	F	2022-2023	FZ-SD	4th Vac	Pfizer	50	2954.79
COV-Flu	18-64 Adult	UGA7-599/CTR-238	62	M	2022-2023	FZ-SD	5th Vac	Pfizer	36	647.65
COV-Flu	18-64 Adult	UGA7-613/CVI-814	62	F	2022-2023	FZ-SD	4th Vac	Pfizer	2	806
COV-Flu	18-64 Adult	UGA7-306/CTR-142	63	F	2022-2023	FZ-SD	5th Vac	Moderna	35	2041.34
COV-Flu	18-64 Adult	UGA8-250	38	F	2023-2024	FZ-SD	Unknown	Pfizer	48	1105.32
COV-Flu	18-64 Adult	UGA8-662/CVI-987	47	F	2023-2024	FZ-SD	Unknown	Pfizer	4	566.97
COV-Flu	18-64 Adult	UGA8-708/CVI-350	51	F	2023-2024	FZ-SD	5th Vac	Pfizer	19	1244.89
COV-Flu	18-64 Adult	UGA8-682/P-258	59	F	2023-2024	FZ-SD	6th Vac	Pfizer	3	314.38
COV-Flu	18-64 Adult	UGA8-352/CTR-028	62	M	2023-2024	FZ-SD	Unknown	Pfizer	16	1967.26
COV-Flu	18-64 Adult	UGA8-384/CTR-031	62	F	2023-2024	FZ-SD	5th Vac	Pfizer	24	3816.74
COV-Flu	65-90 Elderly	UGA6-379/CTR-185	65	F	2021-2022	FZ-HD[g]	3rd Vac	Pfizer	2	871.88354
COV-Flu	65-90 Elderly	UGA6-369/CTR-167	67	M	2021-2022	FZ-HD	3rd Vac	Pfizer	8	20.0
COV-Flu	65-90 Elderly	UGA6-295	68	F	2021-2022	FZ-HD	3rd Vac	Pfizer	5	227.55818
COV-Flu	65-90 Elderly	UGA6-296	68	M	2021-2022	FZ-HD	3rd Vac	Pfizer	5	1940.85523
COV-Flu	65-90 Elderly	UGA6-481	69	F	2021-2022	FZ-HD	3rd Vac	Moderna	10	7352.96322
COV-Flu	65-90 Elderly	UGA6-276/CTR-138	70	F	2021-2022	FZ-HD	3rd Vac	Pfizer	3	1377.3465
COV-Flu	65-90 Elderly	UGA6-294	71	F	2021-2022	FZ-HD	3rd Vac	Pfizer	3	175.99174
COV-Flu	65-90 Elderly	UGA6-308/CVI-967	73	M	2021-2022	FZ-HD	3rd Vac	Pfizer	9	487.47381
COV-Flu	65-90 Elderly	UGA6-353	74	M	2021-2022	FZ-HD	3rd Vac	Pfizer	8	1048.09909
COV-Flu	65-90 Elderly	UGA6-362/CTR-117	77	M	2021-2022	FZ-HD	3rd Vac	Pfizer	13	644.42681
COV-Flu	65-90 Elderly	UGA7-309/CVI-910	66	F	2022-2023	FZ-HD	Unknown	Moderna	12	1410.46
COV-Flu	65-90 Elderly	UGA7-354	66	M	2022-2023	FZ-HD	4th Vac	Pfizer	24	122.69
COV-Flu	65-90 Elderly	UGA7-323/CTR-151	68	F	2022-2023	FZ-HD	Unknown	Pfizer	17	2192.3
COV-Flu	65-90 Elderly	UGA7-369/CTR-167	68	M	2022-2023	FZ-HD	4th Vac	Pfizer	3	125.6262752
COV-Flu	65-90 Elderly	UGA7-304/CVI-989	69	F	2022-2023	FZ-HD	4th Vac	Pfizer	25	2517.04
COV-Flu	65-90 Elderly	UGA7-098/CTR-042	72	M	2022-2023	FZ-HD	5th Vac	Pfizer	20	476.88
COV-Flu	65-90 Elderly	UGA7-320/CTR-154	72	F	2022-2023	FZ-HD	3rd Vac	Pfizer	14	1007.91
COV-Flu	65-90 Elderly	UGA7-148/CVI-966	74	M	2022-2023	FZ-HD	4th Vac	Pfizer	2	78.96
COV-Flu	65-90 Elderly	UGA7-177/CTR-118	74	M	2022-2023	FZ-HD	5th Vac	Pfizer	19	199.53
COV-Flu	65-90 Elderly	UGA7-362/CTR-117	78	M	2022-2023	FZ-HD	4th Vac	Pfizer	2	6070.35

COV-Flu	65-90 Elderly	UGA7-376/CTR-075	81	M	2022-2023	FZ-HD	3rd Vac	Moderna	1	394.2599503
COV-Flu	65-90 Elderly	UGA8-306/CTR-142	65	F	2023-2024	FZ-HD	6th Vac	UNKNOWN	7	598.88
COV-Flu	65-90 Elderly	UGA8-304/CVI-989	69	F	2023-2024	FZ-HD	6th Vac	Moderna	14	909.09
COV-Flu	65-90 Elderly	UGA8-276/CTR-138	72	F	2023-2024	FZ-HD	4th Vac	Pfizer	9	583.13
COV-Flu	65-90 Elderly	UGA8-170/CTR-090	73	F	2023-2024	FZ-HD	Unknown	Pfizer	20	363.66
COV-Flu	65-90 Elderly	UGA8-169/CTR-089	80	M	2023-2024	FZ-HD	6th Vac	Pfizer	20	584.33
COV-Fluc	18-64 Adult	FB2-062/CVI-635	41	M	2022-2023	FB[h]	4th Vac	Pfizer	20	946.30891
Mono-COVID[i]	18-64 Adult	CVI-582	19	F	2021-2022	-	3rd Vac	Pfizer	-	76.08
Mono-COVID	18-64 Adult	CVI-199	22	F	2021-2022	-	3rd Vac	Pfizer	-	263.71
Mono-COVID	18-64 Adult	CVI-419	25	F	2021-2022	-	3rd Vac	Moderna	-	520.63
Mono-COVID	18-64 Adult	CVI-661	26	M	2021-2022	-	3rd Vac	Moderna	-	2306.53
Mono-COVID	18-64 Adult	CVI-423	27	M	2021-2022	-	3rd Vac	Pfizer	-	144.23
Mono-COVID	18-64 Adult	CVI-667	28	F	2021-2022	-	3rd Vac	Pfizer	-	735.07
Mono-COVID	18-64 Adult	CVI-337	29	F	2021-2022	-	3rd Vac	Pfizer	-	906.12
Mono-COVID	18-64 Adult	CVI-486	31	M	2021-2022	-	3rd Vac	Pfizer	-	407.04
Mono-COVID	18-64 Adult	CVI-705	36	F	2021-2022	-	3rd Vac	Pfizer	-	1206.97
Mono-COVID	18-64 Adult	CVI-427	37	F	2021-2022	-	3rd Vac	Pfizer	-	2593.4
Mono-COVID	18-64 Adult	CVI-020	38	M	2021-2022	-	3rd Vac	Moderna	-	576.79
Mono-COVID	18-64 Adult	CVI-316	39	M	2021-2022	-	3rd Vac	Pfizer	-	870.04
Mono-COVID	18-64 Adult	CVI-399	49	F	2021-2022	-	3rd Vac	Pfizer	-	490.85
Mono-COVID	18-64 Adult	CVI-783	52	F	2021-2022	-	3rd Vac	Pfizer	-	1685.42
Mono-COVID	18-64 Adult	CVI-670	55	F	2021-2022	-	3rd Vac	Pfizer	-	371.55
Mono-COVID	18-64 Adult	CVI-295	56	M	2021-2022	-	3rd Vac	Moderna	-	2984.04
Mono-COVID	18-64 Adult	STM-098	56	F	2021-2022	-	3rd Vac	Pfizer	-	529.08
Mono-COVID	18-64 Adult	P-101	57	F	2021-2022	-	3rd Vac	Pfizer	-	20.0
Mono-COVID	18-64 Adult	P-198	60	F	2021-2022	-	3rd Vac	Pfizer	-	1632.34
Mono-COVID	18-64 Adult	CVI-758	61	M	2021-2022	-	3rd Vac	Pfizer	-	405.32
Mono-COVID	18-64 Adult	STM-012	61	F	2021-2022	-	3rd Vac	Pfizer	-	2228.88
Mono-COVID	18-64 Adult	CVI-418	62	F	2021-2022	-	3rd Vac	Moderna	-	1313.45
Mono-COVID	18-64 Adult	CVI-477	62	M	2021-2022	-	3rd Vac	Pfizer	-	2332.14

Mono-COVID	18-64 Adult	P-128	62	F	2021-2022	-	3rd Vac	Pfizer	-	4557.54
Mono-COVID	18-64 Adult	CVI-186	26	F	2022-2023	-	3rd Vac	Pfizer	-	3535.56
Mono-COVID	18-64 Adult	CVI-348	26	M	2022-2023	-	3rd Vac	Moderna	-	5138.54
Mono-COVID	18-64 Adult	CVI-565	26	M	2022-2023	-	3rd Vac	Pfizer	-	3545.02
Mono-COVID	18-64 Adult	CVI-826	29	F	2022-2023	-	3rd Vac	Pfizer	-	631.92
Mono-COVID	18-64 Adult	CVI-778	30	F	2022-2023	-	3rd Vac	Pfizer	-	295.8
Mono-COVID	18-64 Adult	CVI-742	33	F	2022-2023	-	3rd Vac	Moderna	-	5313.5
Mono-COVID	18-64 Adult	CVI-536	38	F	2022-2023	-	3rd Vac	Pfizer	-	686.84
Mono-COVID	18-64 Adult	CVI-908	39	F	2022-2023	-	3rd Vac	Pfizer	-	22.11
Mono-COVID	18-64 Adult	CVI-266	51	F	2022-2023	-	4th Vac	Pfizer	-	338.82
Mono-COVID	18-64 Adult	CTR-233	60	F	2022-2023	-	4th Vac	Moderna	-	451.9
Mono-COVID	18-64 Adult	CVI-367	61	F	2022-2023	-	3rd Vac	Moderna	-	3578.33
Mono-COVID	18-64 Adult	CVI-803	63	F	2022-2023	-	4th Vac	Moderna	-	612.01
Mono-COVID	18-64 Adult	CVI-596	64	F	2022-2023	-	3rd Vac	Pfizer	-	1591.7
Mono-COVID	18-64 Adult	CVI-802	64	M	2022-2023	-	4th Vac	Moderna	-	755.52
Mono-COVID	18-64 Adult	CVI-809	64	F	2022-2023	-	3rd Vac	Pfizer	-	3459.89
Mono-COVID	18-64 Adult	CVI-703	48	F	2023-2024	-	6th Vac	Moderna	-	2636.07
Mono-COVID	65-90 Elderly	CVI-334	65	M	2021-2022	-	3rd Vac	Moderna	-	2188.06
Mono-COVID	65-90 Elderly	CVI-341	67	F	2021-2022	-	3rd Vac	Pfizer	-	297.99
Mono-COVID	65-90 Elderly	CVI-464	67	F	2021-2022	-	3rd Vac	Moderna	-	744.98
Mono-COVID	65-90 Elderly	CVI-619	67	F	2021-2022	-	3rd Vac	Moderna	-	1147.37
Mono-COVID	65-90 Elderly	CVI-372	70	M	2021-2022	-	3rd Vac	Pfizer	-	140.6
Mono-COVID	65-90 Elderly	VTH-028	70	F	2021-2022	-	3rd Vac	Pfizer	-	1991
Mono-COVID	65-90 Elderly	CVI-351	71	F	2021-2022	-	3rd Vac	Pfizer	-	1235.36
Mono-COVID	65-90 Elderly	CVI-508	71	M	2021-2022	-	3rd Vac	Pfizer	-	242.33
Mono-COVID	65-90 Elderly	CVI-573	71	M	2021-2022	-	3rd Vac	Pfizer	-	562.56
Mono-COVID	65-90 Elderly	CVI-651	71	F	2021-2022	-	3rd Vac	Moderna	-	319.18
Mono-COVID	65-90 Elderly	CVI-608	72	F	2021-2022	-	3rd Vac	Pfizer	-	604.67
Mono-COVID	65-90 Elderly	CVI-465	73	M	2021-2022	-	3rd Vac	Moderna	-	4810.32
Mono-COVID	65-90 Elderly	CVI-583	73	F	2021-2022	-	3rd Vac	Pfizer	-	936.41

Mono-COVID	65-90 Elderly	CTR-203	80	M	2021-2022	-	3rd Vac	Pfizer	-	1903.6
Mono-COVID	65-90 Elderly	CVI-335	83	M	2021-2022	-	3rd Vac	Moderna	-	2009.55
Mono-COVID	65-90 Elderly	CVI-289	68	F	2022-2023	-	4th Vac	Moderna	-	1971.21
Mono-COVID	65-90 Elderly	CVI-801	70	M	2022-2023	-	4th Vac	Pfizer	-	4774.53
Mono-COVID	65-90 Elderly	CVI-841	71	F	2022-2023	-	4th Vac	Moderna	-	1438.91
Mono-COVID	65-90 Elderly	CVI-644	72	M	2022-2023	-	4th Vac	Pfizer	-	260.39
Mono-COVID	65-90 Elderly	CVI-608-2	73	F	2022-2023	-	4th Vac	Pfizer	-	1694.54
Mono-COVID	65-90 Elderly	CVI-262	75	F	2022-2023	-	4th Vac	Pfizer	-	461.95
Mono-COVID	65-90 Elderly	CVI-499	75	M	2022-2023	-	4th Vac	Pfizer	-	3198.23
Mono-COVID	65-90 Elderly	CVI-622	78	F	2022-2023	-	3rd Vac	Moderna	-	2458.39
Mono-COVID	65-90 Elderly	CVI-623	80	M	2022-2023	-	3rd Vac	Moderna	-	871.75
Mono-COVID	65-90 Elderly	CVI-374	81	F	2022-2023	-	4th Vac	Moderna	-	204.04
Mono-COVID	65-90 Elderly	CVI-369	82	F	2022-2023	-	4th Vac	Pfizer	-	41.91
Mono-COVID	65-90 Elderly	CVI-370	83	M	2022-2023	-	4th Vac	Pfizer	-	4376.96

[a]UGA/SPARTA ID: University of Georgia/ SARS Sero-Prevalence and Respiratory Tract Assessment Identification. [b]Wuhan Hu-1 PsVN IC50 RLU: pseudovirus neutralization 50% inhibitory concentration in relative luminescence units. [c]COV-Flu: COVID-19 mRNA and seasonal influenza vaccines. [d]F: Female. [e]FZ-SD: Fluzone standard dose. [f]M: Male. [g]FZ-HD: Fluzone high-dose. [h]FB: FluBlok. [i]Mono-COVID: mRNA COVID-19 vaccine only.
